# Expanding the attack surface: Robust profiling attacks threaten the privacy of sparse behavioral data

**DOI:** 10.1126/sciadv.abl6464

**Published:** 2022-08-19

**Authors:** Arnaud J. Tournier, Yves-Alexandre de Montjoye

**Affiliations:** ^1^ Department of Computing, Imperial College London, London, UK.; ^2^ Data Science Institute, Imperial College London, London, UK.

## Abstract

Behavioral data, collected from our daily interactions with technology, have driven scientific advances. Yet, the collection and sharing of this data raise legitimate privacy concerns, as individuals can often be reidentified. Current identification attacks, however, require auxiliary information to roughly match the information available in the dataset, limiting their applicability. We here propose an entropy-based profiling model to learn time-persistent profiles. Using auxiliary information about a single target collected over a nonoverlapping time period, we show that individuals are correctly identified 79% of the time in a large location dataset of 0.5 million individuals and 65.2% for a grocery shopping dataset of 85,000 individuals. We further show that accuracy only slowly decreases over time and that the model is robust to state-of-the-art noise addition. Our results show that much more auxiliary information than previously believed can be used to identify individuals, challenging deidentification practices and what currently constitutes legally anonymous data.

## Teaser

Individuals can be accurately identified in seemingly anonymous behavioral datasets using data collected at a different time.

## INTRODUCTION

Over 22 billion connected devices, from smartphones and wearables to Internet of Things devices, passively collect fine-grained behavioral data about our lives (*1*). The location of a mobile phone is, for instance, collected up to 14,000 times a day (*2*), while a car generates up to 25 gigabytes of data every hour (*3*). These data are widely used. Location data, for example, are used by banks to detect fraudulent behavior (*4*) and predict the likelihood of loan repayment (*5*). They are also used by governments to monitor employment (*6*), quickly respond to natural disasters (*7*), and recently to respond to the coronavirus disease 2019 (COVID-19) pandemic (*8*). Last, researchers have used location data to better understand the spread of infectious diseases (*9*–*11*) or the segregation in cities (*12*). While extremely useful, behavioral data are also extremely personal and sensitive (*13*), as shown by the Cambridge Analytica affair (*14*) and Edward Snowden’s revelations (*15*). In recent surveys, over 80% of Americans (*16*) and 80% of Britons (*17*) have expressed concerns over how their data are used and shared.

Finding a balance between using behavioral data for good and protecting people’s privacy often relies on anonymizing the data. Once anonymized, behavioral data fall outside the scope of data protection laws and can be freely used and shared. In the European Union’s General Data Protection Regulation (*18*) (GDPR, recital 26), data are considered anonymized when “rendered anonymous in such a manner that the data subject is not or no longer identifiable. [ … ] To ascertain whether means are reasonably likely to be used to identify the natural person, account should be taken of all objective factors, such as the costs of and the amount of time required for identification, taking into consideration the available technology at the time of the processing and technological developments.” Similar definitions are found in privacy laws around the world, e.g., in the California Consumer Privacy Act section 1798.140 (h) (*19*) and in new bills currently under examination across the United States [e.g., Washington Senate Bill 5062 section 101 (*20*), Massachusetts Bill HD.3847 (*21*), and Virginia House Bill 2307 section 59.1-571 (*22*)].

Data matching has long been used to reidentify individuals in deemed-anonymous datasets using, e.g., record linkage algorithms (*23*–*25*). The seminal reidentification of the Governor of Massachusetts William Weld’s health records (*26*) and the recent reidentification of President Trump’s tax records (*27*) are examples of high-profile data matching attacks (*28*–*32*). More recently, data matching attacks have been developed and deployed against high-dimensional datasets, such as public transport smart cards (*33*), credit cards (*34*), cryptocurrency transactions (*35*), personal vehicles GPS (*36*), mobile phone mobility (*37*), smartphone application usage (*38*), web histories (*39*–*41*), smart meter data (*42*), and social network graphs (*43*, *44*). New techniques have also been developed to identify individuals from imperfect matches (*41*, *45*), evaluate the correctness of matches (*46*), and assess the robustness of matching in large datasets (*47*).

Matching attacks, however, require the attacker to have access to auxiliary information about the target, which are also available in the dataset. For example, Gov. Weld was identified by his date of birth, gender, and zip code (*26*). These pieces of information were both public and available in the anonymized medical dataset. Assuming that the same information is both available in the dataset and as auxiliary information is, in general, a reasonable requirement for traditional tabular data. For behavioral data, however, this means that auxiliary information about the target and data points in the dataset have to be collected not only over the same period of time but also roughly at the same times. This is a strong requirement that can substantially limit the availability of matching auxiliary information, in particular, when the data are sparse (*45*, *48*). This has led some to question the practical risks posed by data matching for behavioral data and ultimately whether data protection laws should apply to pseudonymized behavioral datasets (*48*–*50*).

Here we present a profiling attack against sparse behavioral data capable of leveraging fully nonmatching auxiliary information, enabling the attacker to use a wide range of auxiliary information including publicly available information. Once trained, our entropy-based model correctly identifies 79% of individuals in a location dataset of 0.5 million people and 93% within a set of 10 candidates. Similarly, on a grocery shopping dataset of 85,000 individuals (*51*), our model correctly identifies 65% and 74% within a set of 10 candidates. Using a metaclassifier, our model reaches an Area Under the Receiver Operating Characteristic (AUROC) of 0.91 and is well calibrated. Our results hold even when (i) the time gap between the dataset and the auxiliary information increases, (ii) state-of-the-art noise is added to the dataset, and (iii) the dataset is large. Together, our results relax a strong requirement of current matching attacks and show that much more auxiliary information than previously thought might be available to reidentify individuals in behavioral datasets. This has broad implications for what constitutes anonymous data in today’s world, challenges current deidentification practices, and emphasizes the need to develop and deploy modern privacy engineering solutions.

## RESULTS

**Fig. 1.**
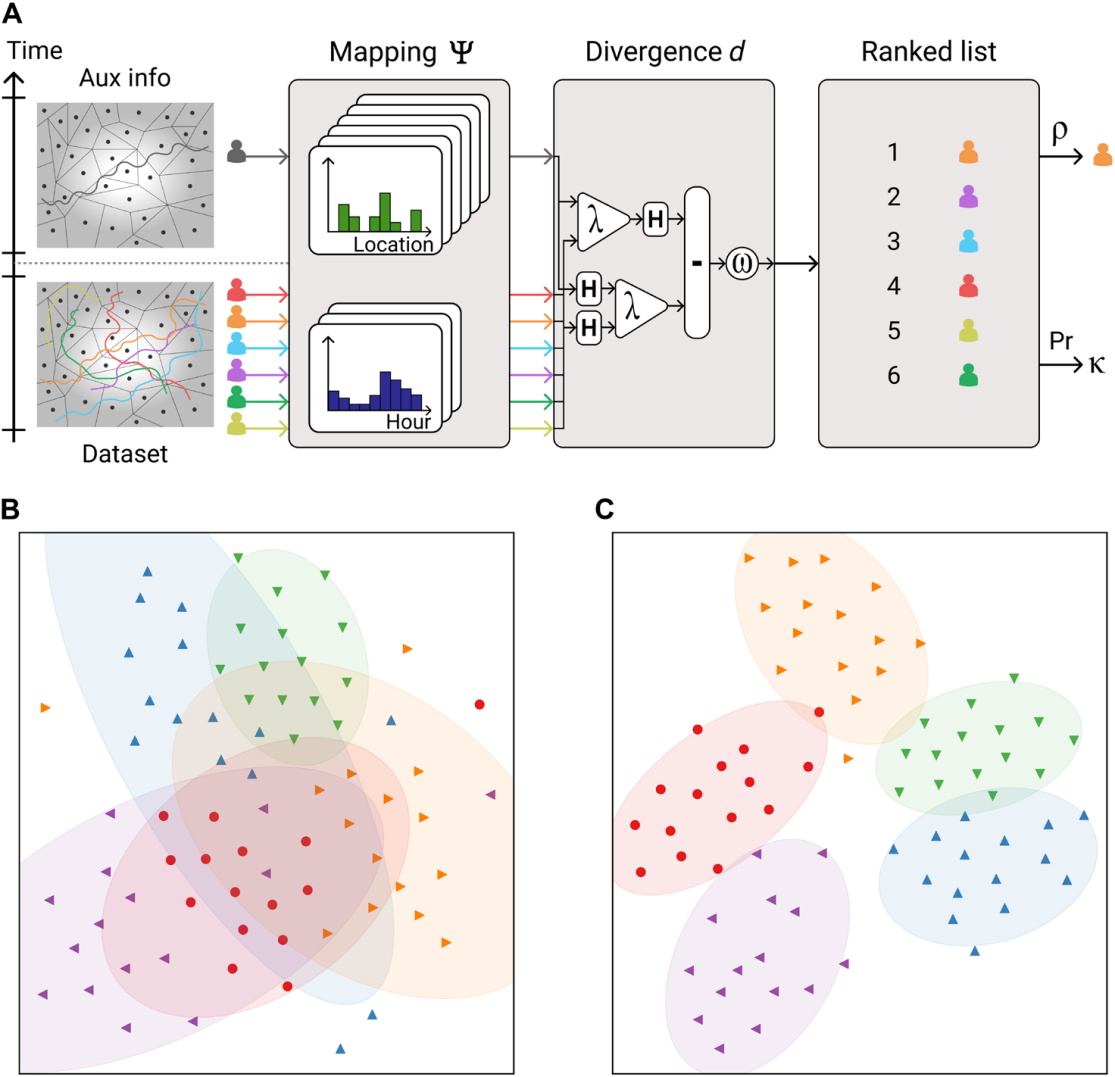
Representation of the attack and effect of training. (**A**) Using auxiliary information τ about the target (top: recorded over 𝒯_aux_), the attacker attempts to identify the target in the dataset Θ_data_ (bottom: recorded over 𝒯_data_). Traces are processed by the model in three steps: (i) Time persistent profiles are computed using Ψ, (ii) the dissimilarities between the auxiliary information and profiles are computed with the divergence *d*, and (iii) potential candidates are ranked and the meta-classifier estimates the likelihood κ of the best candidate ρ to be the target. (**B** and **C**) Representation of profiles built by the model before (B) and after (C) training using *t*-distributed stochastic neighbor embedding (*t*-SNE) on *d* (*97*). Each point is a profile computed from 1 week of location data for a person. The training procedure here improves the ability of our model to distinguish the profiles of a single individual from the profiles of other individuals.

We consider a population *I*_data_ of *N* individuals interacting with a service over a time period $T_{\text{data}}=[t_{\text{data}},t_{\text{data}}^{\prime })$. The service collects a behavioral dataset Θ_data_ = {*y_i_*∣*i* ∈ *I*_data_} where, for each individual *i* ∈ *I*_data_, *y_i_* = ((*t*_*i*,1_, *x*_*i*,1_), …, (*t*_*i*,*n_i_*_, *x*_*i*,*n_i_*_)) is a trace of data points. *x* ∈ 𝒳 (e.g., a physical location) and points are time-ordered (*t*_*i*,1_ ≤ ⋯ ≤ *t*_*i*,*n_i_*_, with *t*_data_ ≤ *t*_*i*,1_ and $t_{i,n_{i}}<t_{\text{data}}^{\prime }$). An attacker holds auxiliary information τ = ((*t*_τ,1_, *x*_τ,1_), …, (*t*_τ,*n*_τ__, *x*_τ,*n*_τ__)) about a target individual *j*, which they hope to use to identify *j* in Θ_data_ (Fig. 1A). The auxiliary information is recorded over a time interval $T_{\text{aux}}=[t_{\text{aux}},t_{\text{aux}}^{\prime })$ (i.e., $t_{\text{aux}}\leq t_{\tau ,1}\leq \cdots \leq t_{\tau ,n_{\tau }}<t_{\text{aux}}^{\prime }$) disjoint from 𝒯_data_ (i.e., $t_{\text{data}}^{\prime }\leq t_{\text{aux}}$ or $t_{\text{aux}}^{\prime }\leq t_{\text{data}}$). We here assume that *j* ∈ *I*_data_ and consider the general case in Discussion.

### Profiling model for sparse data

We consider a space of profiles 𝒮 and a map Ψ from raw traces to profiles. Profiles aim to capture information about an individual that is both specific to that individual and stable over time. Formally, with few assumptions about the behavior of individuals, Ψ performs nonparametric density estimation of *q* random variables extracted from each trace (e.g., location or time of the day). The space of profiles $S=\prod_{k=1}^{q}S_{k}$ is a product of subsets of ${\mathbb{R}}_{k}^{a}$, where *a_k_* is the dimension corresponding to the *k*th variable (see Materials and Methods).

We propose an asymmetric dissimilarity function *d* on 𝒮 to compare the profiles of individuals in the dataset with the auxiliary information available to the attacker

$$\forall X,Y\in S\;d_{\Omega ,\Lambda }(X∥Y)=\sum_{k=1}^{q}\Omega_{k}d_{\Lambda_{k}}(X_{k}∥Y_{k})$$ (1)

where

$$d_{\Lambda_{k}}(X_{k}∥Y_{k})=H(M_{\Lambda_{k}}(X_{k},Y_{k}))-h_{\Lambda_{k}}(X_{k},Y_{k})$$ (2)

The model parameters $\Omega \in {\mathbb{R}}_{+}^{q}$ and Λ ∈ (0,1)*^q^* are shared across individuals. *H* is the information entropy function, and *M*_Λ*_k_*_(*X_k_*, *Y_k_*) = Λ*_k_X_k_* + (1 − Λ*_k_*)*Y_k_* and *h*_Λ*_k_*_(*X_k_*, *Y_k_*) = Λ*_k_H*(*X_k_*) + (1 − Λ*_k_*)*H*(*Y_k_*) are convex combinations (see Materials and Methods). These convex combinations adjust *H* nonnegative gaps of concavity in Eq. 2. The gaps are then combined linearly in Eq. 1 with Ω controlling their respective weights. Gaps capture the amount of statistical uncertainty that would be introduced by mixing profiles *X* and *Y*. Mixing profiles from a single individual is expected to introduce less uncertainty than mixing profiles from distinct individuals, leading to smaller values for *d* (see the Supplementary Materials).

Using the divergence *d*, traces in the dataset Θ_data_ are ranked according to their similarity with the auxiliary information τ. In particular, our model finds the most similar trace $\rho (\tau )=\underset{y\in \Theta_{\text{data}}}{\text{arg}\;\text{min}}\;d\;(\Psi (\tau )\|\Psi (y))$. Once ρ(τ) is found, a meta-classifier using the “second-over-first” score (*41*, *45*) estimates the likelihood ${\hat{\kappa }}_{\tau }$ of ρ(τ) to be correct (see Materials and Methods).

$$s(\tau )=\delta (\tau ,\rho_{2}(\tau ))-\delta (\tau ,\rho_{1}(\tau ))$$ (3)

for δ(τ, ρ*_k_*(τ)) = *d*_Ω,Λ_(Ψ(τ)∥Ψ(ρ*_k_*(τ))), with *k* ∈ {1,2}, ρ_1_(τ) = ρ(τ), and ρ_2_(τ) the second most similar trace to τ.

We train our model using a contrastive loss function, a well-known approach in image representation learning (*52*–*54*). Training traces are split over two disjoint subintervals of 𝒯_data_ into Θ_1_ and Θ_2_ (see Materials and Methods). Traces in Θ_1_ can be viewed as anchors, compared to positive and negative examples in Θ_2_. Formally, for 𝒜 = {(Ψ(*y*_1_), Ψ(*y*_2_)) ∣ *y*_1_ ∈ Θ_1_, *y*_2_ ∈ Θ_2_, *y*_1_ ≡ *y*_2_}, a set of training profiles computed from Θ_1_ and Θ_2_, where *y*_1_ ≡ *y*_2_ indicates two traces originating from the same individual

$$L(\Omega ,\Lambda )=D_{-}(A)-\alpha D_{+}(A)$$ (4)

where

$$D_{-}(A)=\sum_{(X_{1},X_{2})\in A}{\left[d(X_{1}∥X_{2})-d(X_{1}∥\sigma (X_{1}))\right]}_{+}$$ (5)

and

$$D_{+}(A)=\sum_{(X_{1},X_{2})\in A}{\left[d(X_{1}∥\sigma (X_{1}))-d(X_{1}∥X_{2})\right]}_{+}$$ (6)

with

$$\sigma (X_{1})=\underset{Y_{2}\in \Psi (\Theta_{2})\backslash X_{2}}{\text{arg}\;\text{min}}\;\;d(X_{1}∥Y_{2})$$ (7)

The terms of 𝒟_+_ are nonzero for couples (*X*_1_, *X*_2_) ∈ 𝒜 where the model correctly finds *X*_2_ to be the most similar profile for *X*_1_ (positive examples). Reciprocally, the terms of 𝒟_−_ are nonzero on couples where *X*_1_ is incorrectly identified (negative examples). Training minimizes 𝒟_−_ while maximizing 𝒟_+_ according to a balancing meta-parameter α > 0 (see Materials and Methods and Fig. 1, B and C).

### Empirical evaluation

We use a large-scale location dataset collected over 24 consecutive weeks for 0.5 million people through Call Detail Records (CDRs). For every interaction (call or text), CDRs typically contain the pseudonyms of the sender, the recipient, an hourly timestamp, the type of interaction (call or text), the duration of the call, as well as the approximate location of both the sender and the recipient. More specifically, for each party, approximate location refers to the antenna the party was connected to when the interaction occurred. To keep our model general, we here only use the location and hourly timestamp information. On average, traces contain 50.70 data points per week. The dataset Θ_data_ and the auxiliary information τ are fully disjoint and recorded respectively over the weeks 𝒯_data_ = [1,11) and 𝒯_aux_ = [11,16). The remaining weeks (i.e., [16,24]) are used in the next section to study the impact of the time gap $g=t_{\text{aux}}-t_{\text{data}}^{\prime }$ between 𝒯_data_ and 𝒯_aux_ on the model accuracy.

We also validate the generality of our model by applying it to a grocery shopping dataset provided by Instacart (*51*). The dataset contains the ordered list of shopping baskets purchased online by each customer during a year. Each data point is a transaction corresponding to the basket of items purchased by a customer at once. The dataset contains approximately 100,000 individuals with at least 10 recorded transactions throughout the year. Recorded transactions include the purchased quantities of each product, the aisle where each product is stored within the shop (such as vegetables and meat), as well as the day of the week and hour of the day when the transaction happened. As this shopping dataset does not contain location information, we apply our model to profile individuals according to what they typically buy (see the Supplementary Materials). Similarly to the setup we use for the location dataset, the auxiliary information used by the model here is also fully nonoverlapping (see the Supplementary Materials).

**Fig. 2.**
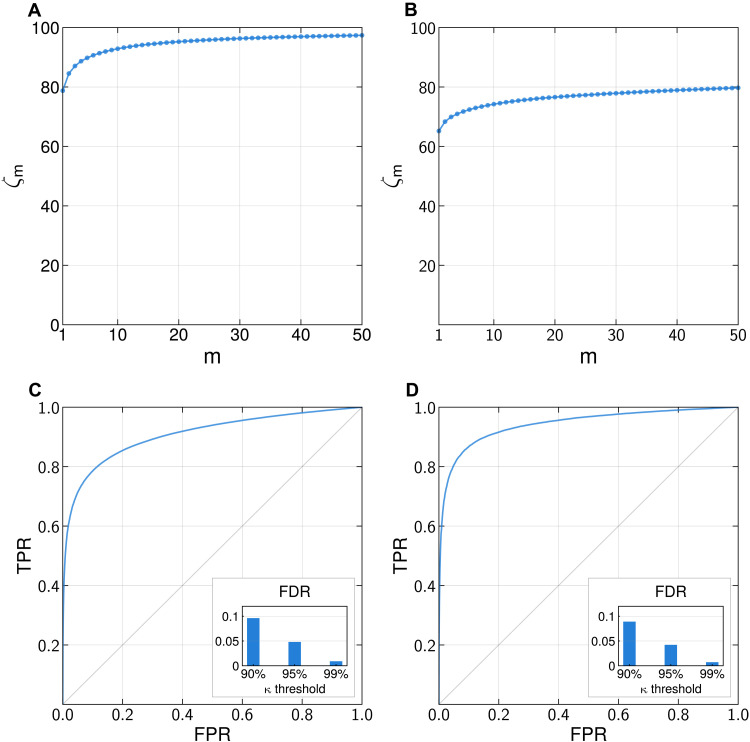
The model identifies the correct individual with high probability. (**A**) Likelihood ζ*_m_* to find a target in the location dataset within the top *m* candidates selected by our model out of *N* = 0.5 million. An attacker has ζ_1_ = 79% chance of correctly identifying the target in the location dataset, with ζ*_m_* increasing rapidly with *m*. (**B**) Likelihood ζ*_m_* to find a target in the grocery shopping dataset within the top *m* candidates selected by our model out of *N* = 85,000 individuals. An attacker has ζ_1_ = 65% chance of correctly identifying the target in the grocery shopping dataset, with ζ*_m_* increasing rapidly with *m*. (**C**) The meta-classifier accurately evaluates whether the individual found by our model in the location dataset is correct (ROC curve, AUC = 0.91). Inset: False discovery rate (FDR) for traces with ${\hat{\kappa }}_{\tau }>\kappa$. For individuals predicted to be correctly found in the location dataset with ${\hat{\kappa }}_{\tau }>0.95$, 4.81% are actually incorrect, showing the method to be well calibrated (see table S4). (**D**) The meta-classifier accurately evaluates whether the individual found by our model in the grocery shopping dataset is correct (ROC curve, AUC = 0.94). Inset: FDR for traces with ${\hat{\kappa }}_{\tau }>\kappa$. For individuals predicted to be correctly found in the grocery shopping dataset with ${\hat{\kappa }}_{\tau }>0.95$, 4.22% are actually incorrect (see table S5). TPR, true positive rate; FPR, false positive rate.

Figure 2A shows that our model has ζ_1_ = 79% chance of correctly finding a target out of *N* = 0.5 million individuals in the location dataset. The attacker would furthermore have ζ_10_ = 93% chance of correctly finding the target in a set of 10 candidates and ζ_50_ = 97.5% chance in a set of 50 candidates, both out of 0.5 million individuals.

Figure 2B shows that our model has ζ_1_ = 65% chance of correctly finding a target out of *N* = 85,000 individuals in the grocery shopping dataset. The attacker would furthermore have ζ_10_ = 74% chance of correctly finding the target in a set of 10 candidates and ζ_50_ = 80% chance in a set of 50 candidates, both out of 85,000 individuals. While a complete analysis of why individuals might be less identifiable in shopping data than location data is beyond the scope of this work, we offer some hypotheses in Discussion.

Figure 2 (C and D) shows that the meta-classifier accurately predicts whether the right individual has been found by our model. It achieves a high AUC [area under the receiver operating characteristic curve (ROC)] of 0.91 for the location dataset (0.94 for the grocery shopping dataset), and the estimated likelihood ${\hat{\kappa }}_{\tau }$ of the right individual to be found is well calibrated. This ensures that an individual found by our model and given a high probability by the meta-classifier is likely to be the right person. For instance, for ${\hat{\kappa }}_{\tau }>0.95$ (respectively 0.9 and 0.99), the empirical likelihood for ρ(τ) to be incorrect (false discovery rate) is 4.85% for the location dataset (respectively 10.4 and 1.0%; see inset in Fig. 2C).

### Time persistence of location profiles

Our behavior is likely to change over time, as we change jobs or partners, move houses, or favor new shops (fig. S4). To evaluate the robustness of profiles against the natural drift of human behavior over time (*55*), we compare the performances of our model when the auxiliary information is collected after a time gap $g=t_{\text{aux}}-t_{\text{data}}^{\prime }$. Starting from *g* = 0, i.e., the previous configuration where the auxiliary information 𝒯_aux_ and the dataset Θ_data_ are collected over disjoint consecutive periods of time 𝒯_data_ = [1,11) and 𝒯_aux_ = [11,16), we increase the time gap *g* and consider auxiliary information collected over 𝒯_aux,g_ = [11 + *g*,16 + *g*).

This experiment shows (fig. S5) that the location profiles built by our model are time persistent, with accuracy ζ_1,*g*_ decreasing slowly over time. For each added week in the time gap, we estimate that ζ_1,*g*_ decreases by 0.93 percentage point on average [±0.29, standard error of the difference (SED), linear fit *R*^2^ = 0.996]. The AUC of the meta-classifier similarly decreases slowly over time (AUC_*g*=0_ = 0.91 down only to AUC_*g*=9_ = 0.90).

To understand why some individuals are more identifiable than others in the location dataset, we compute a handful of summary statistics for each individual and use them in a post hoc analysis with individuals being split into two groups according to their respective identification rate when *g* increases (see Materials and Methods). We found (fig. S5) that individuals that are more identifiable visit more unique locations (30 versus 21 medians, *P* < 10^−15^), that their traces contain more geographical information (geographical entropy of traces: 2.8 versus 2.2 bits of information, *P* < 10^−15^), that they spend most of their time within a small geographical region (radius of gyrations (*56*): 19.8 versus 21.8 km, *P* < 10^−15^), and that they live in less densely populated area (area of the primary Voronoi cell: 7.2 versus 9.6 km^2^, *P* < 10^−15^). These differences suggest that the lifestyle of an individual affects its identifiability in profiling attacks.

### Robustness to noise addition

Noise addition has long been used as a mechanism to prevent identification. Geo-indistinguishability, a technique inspired by Differential Privacy (*57*), has become a popular noise addition mechanism for high-dimensional location data. It has, for instance, been implemented by browser apps such as Location Guard (*58*) and Geoprivacy (*59*). Geo-indistinguishability is achieved by adding, to each data point, independent spatial noises sampled from a bidimensional Laplace distribution with mean radius $\bar{r}=\frac{2}{\epsilon }$ for a given parameter ϵ. Typical values of ϵ used in the literature range from 0.023 m^−1^ ($\bar{r}=100$ m) to 0.0034 m^−1^ ($\bar{r}=600$ m) (*60*–*65*). Knowing the noisy location of a data point thus only reveals a 95% confidence region about its real location with radius ranging from *r*_95_ = 237 m ($\bar{r}=100$m) to *r*_95_ = 1432 m ($\bar{r}=600$m).

**Fig. 3.**
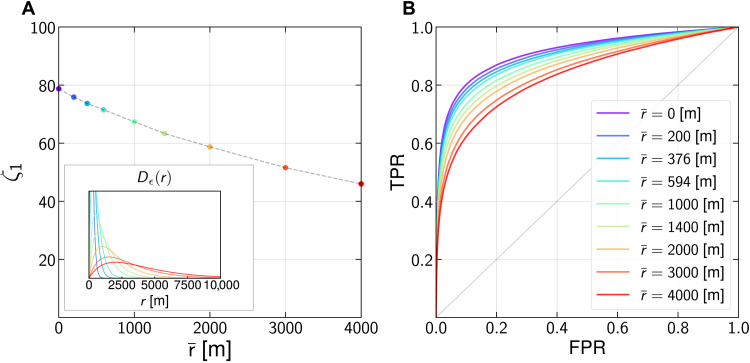
The model is robust to noise addition. (**A**) ζ_1_ when locations in Θ_data_ are perturbed with Laplacian noises. Standard amounts of noises (average radius $\bar{r}<600m$, *r*_95_ < 1432 m) only decrease ζ_1_ by 7 percentage points. For large amounts of noise, ζ_1_ only slowly decreases further, e.g., ζ_1_ = 59% for $\bar{r}=2000m$ (*r*_95_ = 4744 m). Inset: Probability density function *D*_ϵ_(*r*) = ϵ^2^*re*^−ϵ*r*^1_*r* > 0_ of the noise radius *r*. The mean radius is $\bar{r}=\frac{1}{\epsilon }$. (**B**) The predictive power of the meta-classifier, captured by the ROC curves, only decreases slowly as the amount of added noise increases. The AUC decreases from 0.91 without noise down to, at most, 0.83 for $\bar{r}=4000m$.

Figure 3 shows that the accuracy of our model on the location dataset only decreases to 78% when small amounts of noise are added ($\bar{r}=100$m, *r*_95_ = 237 m) and 71% for large amounts of noises ($\bar{r}=600$ m, *r*_95_ = 1432 m). This shows that our model is robust to even the large amounts of spatial Laplace noise addition used in the literature and industry. Even the addition of very large amounts of noise ($\bar{r}>2000$ m) only decreases the accuracy of the model slightly below 60%. This decrease is, however, also likely to strongly affect the utility of the data. $\bar{r}=2000$m indeed means *r*_95_ = 4744 m (see probability density functions in inset Fig. 2C). For comparison, the average area of a zip code in New York City corresponds to a circular region of radius 1300 m.

In this work, we show for the first time how profiling attacks are possible at large scale against sparse behavioral datasets. Profiling attacks relax a strong requirement of matching attacks, especially against behavioral data: the need for auxiliary information to be recorded not only over the same time period but also roughly at the same times. Our attack significantly expands the attack surface by making a much wider range of auxiliary information usable for reidentification, even against noisy datasets. Further research in profiling attacks is likely to lead to even more powerful models. Our results emphasize the need to account for profiling attacks when evaluating what constitutes anonymous data, for instance, the European Union Article 29 WP linkability criteria (*66*) interpretation of GDPR. Technically, our results emphasize the need for formal privacy guarantees and technical privacy engineering solutions enabling the truly anonymous use of behavioral data.

## DISCUSSION

### Scalability of the attack to larger datasets

The size of a dataset is likely to affect the likelihood of a person to be identified in it, with accuracy likely to be lower in larger datasets on average (*46*, *47*). We here study the accuracy of our attack as a function of *N*, the number of individuals in the dataset Θ_data_, assuming everything else is kept equal (see the Supplementary Materials).

**Fig. 4.**
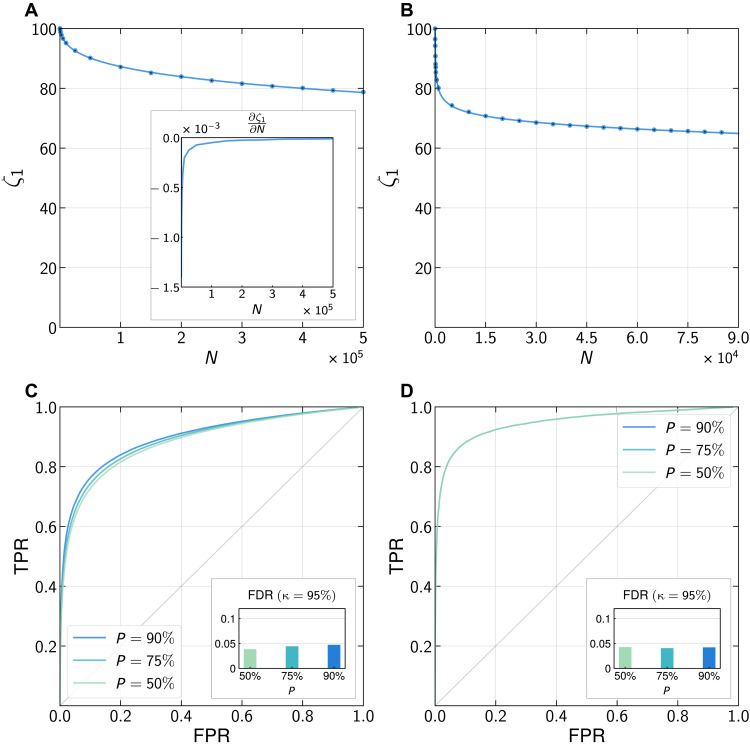
Robustness of the attack to larger datasets and relaxation of the membership assumption. (**A**) Model accuracy ζ_1_ averaged over 10 runs for the location dataset (see table S8). ζ_1_ decreases slowly with population size (log fit, *R*^2^ = 0.999). Inset: The first derivative $\frac{\partial \zeta_{1}}{\partial N}$ converges to zero as *N* increases, confirming that the decrease of ζ_1_ is convex and slow. (**B**) Model accuracy ζ_1_ averaged over 10 runs for the grocery shopping dataset (see table S9). ζ_1_ decreases slowly with population size (log fit, *R*^2^ = 0.99). (**C**) The attack is robust to a relaxation of the membership assumption (location dataset). The prior *P* is the Jaccard index between *I*_aux_ and *I*_data_. Inset: FDR for predictions ρ(τ) estimated to be correct with ${\hat{\kappa }}_{\tau }>0.95$. The likelihood for ρ(τ) to be incorrect is 4.6% for *P* = 0.9 (resp. 4.3% for *P* = 0.75 and 3.3.% for *P* = 0.5), showing that the method is well calibrated even when the membership assumption is relaxed (see table S6). (**D**) The attack is robust to a relaxation of the membership assumption (grocery shopping dataset). The ROC curves for various prior *P* are indistinguishable. Inset: FDR for predictions ρ(τ) estimated to be correct with ${\hat{\kappa }}_{\tau }>0.95$. The likelihood for ρ(τ) to be incorrect is 4.22% for *P* = 0.9 (resp. 4.06% for *P* = 0.75 and 4.27% for *P* = 0.5), showing that the method is well calibrated even when the membership assumption is relaxed (see table S7).

Figure 4 (A and B) shows that the accuracy ζ_1_ of our attack only decreases slowly with *N* for both location and grocery shopping datasets. The first derivative $\frac{\partial \zeta_{1}}{\partial N}$ is strictly increasing, showing that ζ_1_ is strongly convex (see inset). Moreover, $\frac{\partial \zeta_{1}}{\partial N}$ converges to 0 rapidly when *N* increases. Last, a simple logarithmic fit (*R*^2^ = 0.999) shows the decrease of ζ_1_ to behave as a third-order polynomial in log (*N*). Together, these findings strongly suggest that the accuracy of our attack would remain high in most practical settings.

### Removing the membership assumption

We have, throughout the article, worked under the assumption that the attacker knew the target to be in the dataset Θ_data_. While we believe that this assumption is reasonable in many practical settings, there are situations where it might not be the case. We thus extend the meta-classifier to the case where the attacker is unsure whether the target is in the dataset.

The meta-classifier is adapted using a prior *P* on the probability of the target to be in the dataset. In the main text, scores were calibrated using two sets of traces obtained over two disjoint periods of time from the same individuals. Here, the prior *P* is used during the calibration phase as a leave-one-out parameter. Each calibration trace in the first set has its counterpart in the second set virtually removed with probability 1 − *P* (see Materials and Methods). *P* is chosen by the attacker on a case per case basis depending on the information available to them. For instance, *P* could be the sampling rate for sampled data or the market share for a company in the country of interest.

Figure 4 (C and D) shows that our classifier accurately predicts whether the right individual has been found for various levels of prior *P*. For each prior, after calibration, we perform the attack on targets from a new set of individuals *I*_aux_ such that *P* = *J*(*I*_aux_, *I*_data_) the Jaccard index between both sets (*67*). The performances of our meta-classifier only decrease slightly with *P* as new targets, not contained in the dataset, are introduced. In particular, for the location dataset, the AUC decreases from AUC_*P*=1_ = 0.91 (main text) to AUC_*P*=0.9_ = 0.89, AUC_*P*=0.75_ = 0.89, and AUC_*P*=0.5_ = 0.88. For predictions ρ(τ) estimated to be correct with ${\hat{\kappa }}_{\tau }>0.95$, the likelihood for ρ(τ) to be incorrect is 4.6% for *P* = 0.9 (resp. 4.3% for *P* = 0.75 and 3.3% for *P* = 0.5, see inset Fig. 4C).

### Comparison with previous works on location data

While most previous works on location data have investigated attacks where matching auxiliary information is available to an attacker (*34*, *37*, *45*, *68*–*70*), a few attacks using nonoverlapping auxiliary information have been proposed and evaluated on small-scale datasets (from 100 to 50,000 individuals) (*71*–*73*). These attacks are based either on Markov chains (*71*, *72*) or on histograms (*73*). We reimplement and compare our work to six of these methods: four based on histograms using the Jensen-Shannon (JS) divergence (*73*), Bhattacharyya (Bhat) distance (*74*), L1 distance (*75*), and cosine distance (*76*) and two based on Markov chains (*71*, *72*). We compare these methods to our approach in three scenarios: (i) no noise added to the dataset, (ii) small amounts of noise added to the dataset ($\bar{r}=200$ m), and (iii) very large amounts of noise added to the dataset ($\bar{r}=2000$ m).

**Fig. 5.**
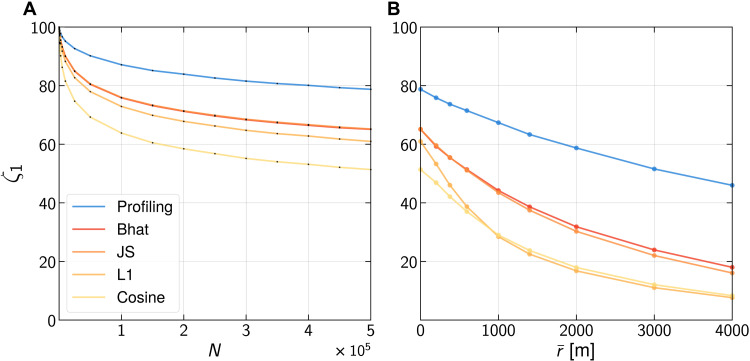
The model outperforms previous work on location data. (**A**) Our model outperforms the baselines at all scales on the location dataset. Accuracies ζ_1_ are averaged over 10 runs (see table S8). (**B**) Our model outperforms the baselines for any amount of added noise, e.g., by 27.6 percentage points for $\bar{r}=3000m$. ζ_1_ also visually decreases more rapidly for baselines than for our model.

Our method outperforms all six previous methods by at least 13 percentage points in each scenario (see table S3). More specifically, we outperform the state of the art by 13.6% in scenario 1, by 16.3% in scenario 2, and by a striking 26.9% in scenario 3. Figure 5 shows how our method outperforms the state of the art (Bhat or JS) across various scales and amounts of added noises. Across the board, histogram-based methods perform better than Markov-based methods (see table S3).

Deep Learning methods have been developed recently to extract representations from raw sequential data, e.g., Contrastive Predictive Coding (*77*), Recurrent Attention Models (*78*), and Autoencoder architectures with Recurrent Neural Networks (*79*) and Autoregressive models (*80*). These representations might, in future work, replace the simple profiles we used here (see Materials and Methods), although questions remain on the applicability of these methods to sparse data. Similarly, future specialized models for behavioral datasets are likely to be developed. Both will ultimately further increase the scope and accuracy of profiling attacks and the risk they pose to our privacy.

### Limitation—auxiliary information

Throughout this work, we evaluate the potential of a person to be identified in a location dataset using fully nonoverlapping auxiliary information coming from the same modality. In some cases, an attacker might try to identify someone using auxiliary information coming from other modalities including publicly available information, such as social media posts, or privately collected information, such as the WiFi connection data used in the recent reidentification and subsequent outing of a U.S. priest (*81*). For ethical, legal (*82*), and contractual reasons, we did not attempt to identify individuals in our dataset using auxiliary information coming from other modalities. Unless the auxiliary information comes from a modality independent from the one used to collect the dataset (e.g., a credit card only used for expenses abroad with mobile phone dataset recorded only in the country), we expect our model to perform well and our results to qualitatively hold.

### Limitation—noise addition mechanism

We here consider, Geo-indistinguishably, a local noise addition mechanism that has traditionally been used for location data (*58*, *59*). A range of other mechanisms could be considered. For instance, one could decide to report the same obfuscated location every time an individual is in a corresponding real location. One could also consider global mechanisms such as *k*-anonymity (*83*). While some of these mechanisms might prove more effective against our attack, something we leave for future work, their impact on the downstream utility of the dataset has to be carefully considered. In particular, the biases introduced by nontruthful methods are considered generally problematic and global methods such as *k*-anonymity have been shown to strongly affect utility (*83*). We are skeptical that behavioral data can be anonymized at individual level while retaining general utility. Instead, we believe modern privacy engineering methods, such as query-based systems (*84*, *85*), and formal guarantees, such as Differential Privacy, to be the way forward when it comes to safely releasing behavioral data.

### Discrepancies between location and grocery shopping datasets

While a complete analysis of why individuals might be less identifiable in shopping data than location data is beyond the scope of this work, we offer some hypotheses below. First, the grocery shopping dataset used here is much sparser than the location dataset (0.52 data points per week on average for the grocery shopping dataset versus 50.70 data points per week on average for the location dataset). This is likely to affect the computation of profiles by density estimation, making them less accurate. Second, grocery shopping data points might be less identifiable than location data points. For instance, groceries online are mostly purchased from the first category page displayed by retailers (*86*), which could reduce the diversity of shopping baskets across individuals. Third, shopping patterns might be less stable over time than location patterns. Previous works have shown human mobility to be fairly predictable, especially with regard to home and work locations (*55*, *87*, *88*). On the other hand, customers seem to shop for groceries online only as a complement to traditional stores (*89*), with many situational factors influencing the loyalty of customers and their purchasing habits over time (*90*).

Last, shopping patterns have been used to train recommender systems. These systems learn to predict future purchases through collaborative filtering, deducing future purchases from what other individuals have purchased in the past. However, while recommender systems learn that customers who bought *X* will also likely be interested in *Y*, our model learns the singularities of the shopping habits of an individual to then identify the specific list of their purchases in the past.

## MATERIALS AND METHODS

Our model is an open-set inductive classifier based on nearest neighbor classification (1-NN) (*91*). Open-set means that classes, i.e., the identities of the individuals in the dataset, are disjoint between training, validation, and testing, thus providing the attacker with a model readily applicable to new individuals. Previous studies have shown that 1-NN performances can be improved by learning the model’s distance (*92*, *93*).

Our methodology contribution can be summarized into three points: (i) We propose an abstract space as input of the model, the space of profiles, and a method to map raw data into that space as collections of histograms. (ii) Within the space of profile, we propose a supervised learning method similar to recent works in distance learning (*92*, *93*) to learn a new divergence to compare profiles. (iii) From the divergence values, we propose a method to estimate the likelihood of auxiliary information to be correctly classified. Our framework, which we will now describe, makes no parametric assumption about the distribution of the data.

### Formalism

Behavioral data are individual-level temporal data containing discrete events characterizing the behavior of each individual. We model the generation of these discrete events as point processes, a general nonparametric model for point pattern analysis (*94*). Formally, for each individual, we consider a trace *y* as a realization of a point process *Y* on T × X, where T ⊂ ℝ_+_ is a time interval over which the data are recorded and $X=\prod_{k}X_{k}$ is a multidimensional space with each *X_k_* either discrete or an interval on the real line. For instance, in the main text, X is the (single-dimensional) finite set of the indexed geographical regions around each antenna. We further assume that these point processes are invariant, as random elements whose values are point patterns, by week translation over time. This is a modeling assumption that works well enough to model the weekly patterns, followed by individuals’ behavior aside from holidays and life-changing events (*55*, *87*, *88*). Under this assumption, *Y* has similar distributions over all T′ × X, where T′ ⊂ T′ is a 1-week time interval.

### Mapping traces to profiles

Using a map Ψ : Θ → S from raw traces to the space of profiles, we compute profiles aiming to capture the recurrent patterns of an individual while reducing the microvariations observed in the data. Profiles are collections of density estimates corresponding to variables obtained from each point process Y*_i_* (see the Supplementary Materials). Formally, for each individual *i*, the profile Ψ(*Y_i_*) = *Z_i_* = (*Z*_*i*,*k*_)_*k*=1, …, *q*_ is a collection of random variables *Z*_*i*,*k*_ taking values on their respective probability simplex S*_k_* (with $S=\prod_{k=1,\ldots ,q}S_{k}$). Here, we choose *Z*_*i*,*k*_ to be a histogram obtained using the random counting measure *N_i_* associated with Y*_i_* on a collection B*_k_* of Borel sets of T × X

$$Z_{i,k}=(\frac{N_{i}(B)}{\sum_{B\prime \in B_{k}}N_{i}(B\prime )})$$ (8)

with *N_i_*(*B*) = # (*B* ∩ *Y_i_*) the random variable counting the number of events of *Y_i_* in *B*, for any *B* ∈ B*_k_*. Aiming for profiles to be time persistent and robust to added noise, we consider collections of Borel sets B*_k_* corresponding to aggregating events time-wise (over 𝒯) and value-wise (over 𝒳) (see the Supplementary Materials). Although beyond the scope of this work, other density estimation methods, e.g., kernel density estimators, could be used for the variables *Z*_*i*,*k*_.

### Divergence

Our model learns how important each random variable *Z*_·,*k*_ is for profiles to be identifiable, and weights these variables accordingly. Each variable is valued on a probability simplex of up to a few thousand dimensions. Weights $\Omega \in {\mathbb{R}}_{+}^{q}$ and Λ ∈ [0,1]*^q^* are, by design, shared across individuals for the model to be inductive. This allows the model to be applied to individuals that are not seen during training and validation.

We define the model divergence $d_{\Omega ,\Lambda }=\sum_{k=1}^{q}\Omega_{k}d_{\Lambda_{k}}$ as a linear combination, weighted by Ω, of pairwise subdivergences of the variables *Z_i_*_,*k*_ on their respective simplexes. More specifically, the subdivergence

$$d_{\Lambda_{k}}(Z_{i,k}∥Z_{i^{\prime },k})=H(M_{i,i^{\prime },k})-h_{i,i^{\prime },k}$$ (9)

compares the convex combination *M*_*i*,*i*^′^,*k*_ = Λ*_k_Z*_*i*,*k*_ + (1 − Λ*_k_*)*Z*_*i*^′^,*k*_ of the histograms *Z*_*i*,*k*_ and *Z*_*i*^′^,*k*_ via the entropy *H* on 𝒮*_k_* (*M*_*i*,*i*^′^,*k*_ ∈ 𝒮*_k_* as probability simplexes arestable by convex combination) to the convex combination *h*_*i*,*i*^′^,*k*_ = Λ*_k_H*(*Z*_*i*,*k*_) + (1 − Λ*_k_*)*H*(*Z*_*i*^′^,*k*_) of the entropies taken separately. Because of the concavity of *H*, for all *k*, the subdivergence *d*_Λ*_k_*_ is valued in ℝ_+_.

### Empirical setup

Figure S1 shows how we split the location dataset to train, validate, test, and calibrate our model. In particular, the dataset is split into training sets Θ_1_ and Θ_2_, validation sets Θ_3_ and Θ_4_, testing sets Θ_data_ and Θ_aux_, and score calibration sets Θ*_A_* and Θ*_B_*.

For testing, traces are collected over $T_{\text{data}}=[t_{\text{data}},t_{\text{data}}^{\prime })$ from individuals in *I*_data_. Auxiliary information is fully nonoverlapping, collected over $T_{\text{aux}}=[t_{\text{aux}},t_{\text{aux}}^{\prime })$ with $t_{\text{data}}^{\prime }\leq t_{\text{aux}}$ from targets in *I*_aux_ such that the Jaccard index between *I*_data_ and *I*_aux_ is equal to the attacker’s prior *P*. For training, traces are collected over a split of T_data_ into two consecutive time intervals T_1_ = [*t*_0_, *t*_1_) (with *t*_0_ = *t*_data_) and T_2_ = [*t*_1_, *t*_2_) (with *t*_2_ − *t*_1_ = *t*_1_ − *t*_0_) from individuals in *I*_train_. Training individuals *I*_train_ are disjoint from *I*_data_ and *I*_aux_. For validation, traces are collected over a split of T_data_ into two other consecutive time intervals T_3_ = [*t*_2_, *t*_3_) and T_4_ = [*t*_3_, *t*_4_) (with $t_{4}=t_{\text{data}}^{\prime }$ and *t*_4_ − *t*_3_ = *t*_3_ − *t*_2_) disjoint from T_1_ and T_2_. Individuals *I*_valid_ used for validation are disjoint from *I*_data_, *I*_aux_, and *I*_train_. Last, to calibrate the scores, traces are recorded over another split of T_data_ into T*_A_* = [*t_A_*, *t_B_*) and T*_B_* = [*t_B_*, *t*_4_) (with $t_{4}-t_{B}=t_{B}-t_{A}=t_{\text{aux}}^{\prime }-t_{\text{aux}}$, i.e., 5 weeks here) from individuals in *I*_data_.

We select a 80/20 split for the time, with 10 weeks of T_data_ split into 4 and 4 weeks for T_1_ and T_2_, and 1 and 1 week for T_3_ and T_4_, all disjoints. We used a small number of individuals for training (10,000) and validation (1000) to illustrate the strength of our model even when tested orders of magnitude above its training size (*N* = 0.5 million). Individuals were kept strictly separate between training, validation, and testing to prevent overfitting and to show the inductive strength of our model.

### Training

Parameters Ω and Λ are obtained by minimizing the average training error using a standard mini-batch Adam stochastic gradient descent (*95*). In particular, a mini-batch θ*_t_* is drawn at each step *t* from traces in Θ_1_ such that #θ_*t*,+_ = # θ_*t*,−_ = 100 individuals, where θ_*t*,+_ = {*y*^1^ ∈ θ*_t_*∣ρ_*t*,Θ_2__(*y*^1^) ≡ *y*^1^} and θ_*t*,−_ = {*y*^1^ ∈ θ*_t_*∣ρ_*t*,Θ_2__(*y*^1^) ≢ *y*^1^} for $\rho_{t,\Theta_{2}}(y^{1})=\underset{y\in \theta }{\text{arg}\;\text{min}}\;\;d_{\Omega_{t},\Lambda_{t}}(\Psi (y^{1})∥\Psi (y))$. This allows balancing positive and negative examples at each step during training, e.g., when the majority of traces in Θ_1_ are positive examples.

$$L(\Omega ,\Lambda )=D(\theta_{-})+\alpha D(\theta_{+})$$ (10)

where

$$D(\theta )=\frac{1}{\#\theta }\sum_{y\in \theta }[d_{\Omega ,\Lambda }(\Psi (y^{1})∥\Psi (y^{2}))-d_{\Omega ,\Lambda }(\Psi (y^{1})∥\Psi (\sigma (y^{1})))]$$ (11)

where σ(*y*^1^) = ρ_Θ_2_∖{*y*^2^}_(*y*^1^). At every iteration, Λ*_t_* is clipped within (0,1)*^p^* and Ω*_t_* above 0. Although we balanced the contribution of positive and negative examples, the values of 𝒟 in Eq. 11 could still be different between these two groups, particularly when noise is added to the dataset. The balance between 𝒟(θ_+_) and 𝒟(θ_−_) is thus controlled by the meta-parameter α > 0 (see figs. S3 and S4). To select α, the validation accuracy after training $\zeta^{\text{val}}(\alpha )=\frac{1}{\#I_{\text{valid}}}\sum_{y^{3}\in \Theta_{3}}1_{\rho_{\Theta_{4}}(y^{3})\equiv y^{3}}$ is maximized by grid search (see the Supplementary Materials).

### Calibrating scores

We compute the likelihood of the right individual to have been found, i.e., of ρ(τ) to be correct, using a standard second-over-first score from the literature (*41*, *45*):

$$s(\tau )=\delta (\tau ,\rho_{2}(\tau ))-\delta (\tau ,\rho_{1}(\tau ))$$ (12)

where δ(*x*, *y*) = *d*(Ψ(*x*)∥Ψ(*y*)), ρ_1_(τ) = ρ(τ), and ρ_2_(τ) is the second best candidate for τ. For any desired minimum likelihood κ of ρ(τ) to be correct, ρ(τ) is rejected if *s*(τ) < *s*^κ^, where *s*^κ^ is a score threshold corresponding to κ calibrated using Θ*_A_* and Θ*_B_*. The likelihood of ρ(τ) to be correct is then estimated by ${\hat{\kappa }}_{\tau }$ such that $s^{{\hat{\kappa }}_{\tau }}=s(\tau )$.

In practice, an attacker will often have a prior about the likelihood of a target to be in the dataset, e.g., because of the market share of a service in a country. We denote *P* the attacker’s prior on the probability of an auxiliary information to correspond to an individual in *I*_data_, matching in our experiments the Jaccard index (*67*) between *I*_aux_ and *I*_data_ (in particular, in the main text *P* = 1). The distribution of scores is then obtained by comparing each trace *y^A^* within Θ*_A_* with all the traces in Θ*_B_*, after the trace corresponding to *y^A^* has been removed from Θ*_B_* with probability 1 − *P*. Formally, for *N* independent Bernoulli variables (*B_i_*(*p*))_*i* ∈ *I*_data__ of parameter *p*, thresholds *s*^κ^ are obtained by solving $\frac{1}{N}\sum_{i\in I_{\text{data}}}(B_{i}\times 1_{\left\{\rho_{\Theta_{B}}(y_{i}^{A})=y_{i}^{B}|s(y^{A})>s^{\kappa }\right\}}+(1-B_{i})\times 1_{\left\{\rho_{\Theta_{B}\setminus \{y_{i}^{B}\}}(y_{i}^{A})=y_{i}^{B}|s(y^{A})>s^{\kappa }\right\}})=\kappa$.

### Determinants of identifiability

To better understand what might make an individual identifiable, we perform a post hoc study of a handful of summary statistics of an individual’s behavior: the radius of gyration of an individual, the area of the main visited antenna cell, the number of unique antenna cells visited, and the entropy of the location distribution over antenna cells. We consider people who are easier to identify to be target individuals that our model manages to correctly identify in at least 50% of our time gap experiments with T_aux,*g*_ (group 1). All remaining targets are considered harder to identify, including the ones that are never correctly identified (group 2). Of the 420,787 targets considered in the time gap experiment, 312,985 are in group 1, 100,425 are in group 2, and the remaining 7377 were discarded for having their main cell on the edge of the map where the area could not be computed (see the Supplementary Materials). To study discrepancies between these two groups, we perform a Kruskal-Wallis test (*96*), a nonparametric test (see fig. S5). Note that while using these summary statistics as additional features for our model might further improve its performances, we do not use them as they might not be transferable to behavioral datasets beyond location data.

### Noise addition

Given a spatiotemporal point (*t*, *x*) ∈ T × ℝ^2^ such that *x* are GPS coordinates, Geo-indistinguishability (*60*) translates (*t*, *x*) spatially to (*t*, *x* + **r**(ϵ)) with a random noise vector of norm *r*(ϵ) drawn according to the density *D*_ϵ_(*r*) = ϵ^2^*re*^−ϵ*r*^1_*r*>0_.

For CDR, adding noise to the GPS coordinate of the antenna routing the call would unfairly dampen the effect of the added noise. The coverage of each antenna indeed forms a Euclidean Voronoi cell around its location, often effectively canceling out the added noise. Instead, for each data point (*t*, *x*) ∈ T × 𝒳 with 𝒳 the set of these cells, we uniformly sample a GPS point (*t*, (𝓁, *L*)) ∈ *T* × ℝ^2^ within the cell *x*. The noisy GPS point (*t*, (𝓁, *L*) + **r**(ϵ)) is then projected to (*t*, *x*_ϵ_) ∈ T × X, with *x*_ϵ_ the cell containing the noisy GPS coordinates (𝓁, *L*) + **r**(ϵ). The sets Θ_data_, Θ_1_, Θ_2_, Θ_3_, Θ_4_, Θ*_A_*, and Θ*_B_* are thus perturbed into $\Theta_{\text{data}}^{\epsilon }$, $\Theta_{1}^{\epsilon }$, $\Theta_{2}^{\epsilon }$, $\Theta_{3}^{\epsilon }$, $\Theta_{4}^{\epsilon }$, $\Theta_{A}^{\epsilon }$, and $\Theta_{B}^{\epsilon }$. Noises are drawn independently for each data point before the model is trained, validated, tested, and calibrated.

## Supplementary Materials

### This PDF file includes:

Sections S1 to S4 Figs. S1 to S6 Tables S1 to S9 References
