# Supplementary material for: Expanding the attack surface: Robust profiling attacks threaten the privacy of sparse behavioral data
Source: Sci Adv. 2022 Aug 19;8(33):eabl6464. doi: 10.1126/sciadv.abl6464 (PMC11323786; doi:10.1126/sciadv.abl6464)
Supplement: Supplementary file 1 — Sections S1 to S4 Figs. S1 to S6 Tables S1 to S9 References [file sciadv.abl6464_sm.pdf]

Science Advances
AAAS

Supplementary Materials for

Expanding the attack surface: Robust profiling attacks threaten the privacy of sparse behavioral data

Arnaud J. Tournier and Yves-Alexandre de Montjoye

Corresponding author: Arnaud J. Tournier, arnaud.tournier@centraliens.net; Yves-Alexandre de Montjoye, demontjoye@imperial.ac.uk

Sci. Adv. **8**, eabl6464 (2022)
DOI: 10.1126/sciadv.abl6464

This PDF file includes:

Sections S1 to S4
Figs. S1 to S6
Tables S1 to S9
References

S1 Datasets

S1.1 Location data

Our results in the main text rely on a standard Call Details Records (CDR) dataset containing > 0.5M individuals with at least one record per day on average during the first 15 weeks of the collection period. CDR data are both temporally and spatially sparse, with each record containing a timestamp with hourly precision and the geographical coordinates of the antenna in the network through which the call or text was routed. Moreover, contrary to GPS data for instance, CDR records contain bursty sequences separated by long gaps of information [56, 98]. Because of these limitations, our results on CDR data are a lower bound of what could be achievable with higher frequency and higher resolution location data. Fig. S1 shows how this dataset is split along the individual and the time dimensions into training, validation, testing, and calibration sets to avoid overfitting.

S1.2 Grocery shopping data

The extension of our model to grocery shopping data relies on the 2017 Instacart dataset [51], containing approximately 100,000 individuals with at least 10 recorded transactions through-

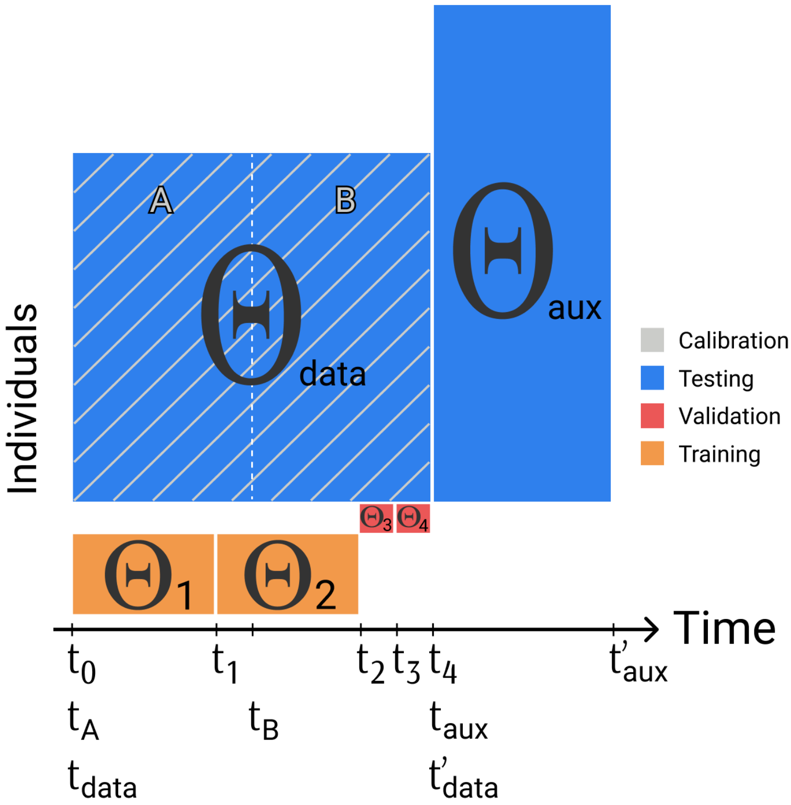

Figure S1: Partitioning of the raw dataset time-wise and individual-wise into training, validation, testing, and calibration sets. Traces are partitioned into training sets (Θ_1 and Θ_2), validation sets (Θ_3 and Θ_4), testing sets (Θ_{aux} and Θ_{data}), and calibration sets (Θ_A and Θ_B). In the main text, $10,000$ individuals were used for training, $1,000$ for validation, and $500,000$ for testing and calibration, all randomly sampled.

out the year. Similarly to the location dataset, this dataset is split along the individual and the time dimensions into training, validation, testing, and calibration sets to avoid overfitting (see Fig. S1). For individuals, we split according to the same rule as for location data: I_{train} (5,000 individuals), I_{valid} (1,000 individuals), and I_{data} (85,000 individuals) are disjoint and randomly sampled from the original dataset. For the time dimension, we cannot split the dataset in the same absolute way as we did for the location data (i.e. using weeks $[1, 11)$ and $[11, 16)$) as data points do not contain timestamps. Instead, the sequence of data points ordered temporally is available for each individual. We thus allocate, for each individual, the first 80% of each sequence to be the data contained in the dataset Θ_{data}, and the remaining 20% to be the external information available to the attacker (Θ_{aux}, fully non-overlapping). In this dataset, each data point is a basket containing the list of items purchased by a customer at once. This includes the purchased quantities of each product, the aisle where each product is stored within the shop (such as vegetables, meat, ...), as well as the day of the week and hour of the day when the transaction happened.

S2 Supplementary Methods

S2.1 Profiles

We use Borel sets that aggregate the point processes $\mathbf{Y}_i$ on $\mathcal{T} \times \mathcal{X}$ over time, in order to obtain recurrent weekly patterns. More specifically, we consider $d : t \in \mathcal{T} \mapsto d(t) \in [0, \ldots, 6]$ the surjective *Day of the Week* function such that, e.g. $d^{-1}(\{5, 6\})$ is the set of all the times within $\mathcal{T}$ corresponding to weekends, and $h : t \in \mathcal{T} \mapsto h(t) \in [0, \ldots, 23]$ the surjective *Hour* function, i.e. $h(t)$ is the hour of the day corresponding to timestamp t. With these notations, the collection $\mathcal{B}_0 = \left(d^{-1}([0, \ldots, 4]) \cap h^{-1}(\{k\}) \times \mathcal{X}\right)_{k \in [0, \ldots, 23]}$, e.g., yields a random variable $Z_{i,0}$ counting during weekdays ($[0, \ldots, 4]$) the number of recorded data points from the process $\mathbf{Y}_i$ within each hour of the day ($[0, \ldots, 23]$).

Location data points are considered in the main text to be generated by point processes $\mathbf{Y}_i$ on $\mathcal{T} \times \mathcal{X}$. On the other hand, each grocery shopping data point is a basket of multiple items purchased at once, potentially with quantities higher than 1. In this case, and more generally for datasets where each data point is a list of records, it can be useful to also consider the point processes generating these records individually. For such dataset, using the $\mathcal{T} \times \mathcal{X}$ notation, $\mathcal{X}$ is the (multi-dimensional) set of purchasable baskets. This means that the elements of $\mathcal{X}$ are multisets, a definition of sets allowing for multiple instances of each element, built over the set of purchasable products $\mathcal{P}$. Instead of seeing them as baskets, each product purchased by a customer i, including each of the multiple instances of the same item, can be seen as a single record generated by a point process $\mathbf{Y}'_i$ on $\mathcal{T} \times \mathcal{P}$. We use both interpretations in what follows, $\mathcal{T} \times \mathcal{X}$ when referring to $\mathbf{Y}_i$, and $\mathcal{T} \times \mathcal{P}$ when referring to $\mathbf{Y}'_i$.

In cases where the attacker has access to information about the structure of the value

space $\mathcal{X}$, the model can also use aggregation over $\mathcal{X}$ similarly to the way we do it for time. In our location dataset, the attacker has access to the GPS coordinates of the antennas, allowing for aggregation of locations using the notion of neighboring antennas. This aggregation could, for instance, help the model understand that an individual is in a given region of the country rather than near a particular antenna. Looking at wider regions than single antenna cells might also help make the model more robust to noise addition. We thus define $f : x \in \mathcal{X} \mapsto f(x) \in \mathcal{P}(\mathcal{X})$ the neighbor function that maps an antenna cell $x \in \mathcal{X}$ to the region $f(x)$ containing x and its neighboring antennas cells. Note that f acts as a convolution filter with constant uniform weights here, with each antenna cell belonging to multiple regions. In our grocery shopping dataset, the attacker has access to the aisle to which each purchased product belongs. The aggregation is here done with a predefined hierarchical structure: for a product $p \in \mathcal{P}$, $f(p)$ is the aisle containing p. Considering aisles instead of products could help the model understand what kind of products an individual buys (e.g. wines).

Table S1 lists the collections of Borel sets used to build profiles for the location dataset. Table S2 lists the collections of Borel sets used to build profiles for the grocery shopping dataset. By design, to keep our model general, we only use very general profiles obtained from non domain-specific information here. Further models could incorporate domain-specific information for CDR data, for example the summary statistics we use for the posthoc analysis or geographical information such as Euclidian distances between visited locations. Similarly, domain-specific information could be extracted from shopping data, e.g. the product prices. Our results should therefore be considered a lower bound on the performances that can be achieved in each case.

Table S1: List of the 12 Borel Collections $\mathcal{B}_1, \ldots, \mathcal{B}_{12}$ used for the location dataset

k	$\mathcal{B}_k$	Description
1	$(d^{-1}([0,\ldots,4]) \times \{x\})_{x\in\mathcal{X}}$	Frequencies of locations recorded during week days
2	$(d^{-1}([5,6]) \times \{x\})_{x\in\mathcal{X}}$	Frequencies of locations recorded during weekends
3	$(\mathcal{T} \times \{x\})_{x\in\mathcal{X}}$	Frequencies of locations overall
4	$(d^{-1}([0,\ldots,4]) \times \{f(x)\})_{x\in\mathcal{X}}$	Frequencies of neighborhoods recorded during week days
5	$(d^{-1}([5,6]) \times \{f(x)\})_{x\in\mathcal{X}}$	Frequencies of neighborhoods recorded during weekends
6	$(\mathcal{T} \times \{f(x)\})_{x\in\mathcal{X}}$	Frequencies of neighborhoods overall
7	$(d^{-1}([0,\ldots,4]) \cap h^{-1}(k) \times \mathcal{X})_{k\in[0,\ldots,23]}$	Hour frequencies recorded over week days
8	$(d^{-1}([5,6]) \cap h^{-1}(k) \times \mathcal{X})_{k\in[0,\ldots,23]}$	Hour frequencies recorded over weekends
9	$(h^{-1}(k) \times \mathcal{X})_{k\in[0,\ldots,23]}$	Hour frequencies overall
10	$(d^{-1}([0,\ldots,4]) \times \mathcal{X})$	Number of records over week days
11	$(d^{-1}([5,6]) \times \mathcal{X})$	Number of records over weekends
12	$(\mathcal{T} \times \mathcal{X})$	Number of records overall

The formula given in the main text to compute the variables $Z_{\cdot,k}$ would actually yield the value 1 for any collection of Borel sets $\mathcal{B}$ where $\#\mathcal{B} = 1$ (such as $\mathcal{B}_{10}$, $\mathcal{B}_{11}$, and $\mathcal{B}_{12}$ here). As this would be of little use, instead of normalizing the counts N_i for these variables as done in that formula, we suggest to divide N_i by a normalizing factor M capturing the

Table S2: List of the 12 Borel Collections $\mathcal{B}_1, \ldots, \mathcal{B}_{12}$ used for the grocery shopping dataset

k	$\mathcal{B}_k$	Description
1	$(d^{-1}([0,\ldots,4]) \times \{p\})_{p\in\mathcal{P}}$	Frequencies of products bought during week days
2	$(d^{-1}([5,6]) \times \{p\})_{p\in\mathcal{P}}$	Frequencies of products bought during week days
3	$(\mathcal{T} \times \{p\})_{p\in\mathcal{P}}$	Frequencies of products bought overall
4	$(d^{-1}([0,\ldots,4]) \times \{f(p)\})_{p\in\mathcal{P}}$	Frequencies of aisles bought from during week days
5	$(d^{-1}([5,6]) \times \{f(p)\})_{p\in\mathcal{P}}$	Frequencies of aisles bought from during weekends
6	$(\mathcal{T} \times \{f(p)\})_{p\in\mathcal{P}}$	Frequencies of aisles bought from overall
7	$(d^{-1}([0,\ldots,4]) \cap h^{-1}(k) \times \mathcal{X})_{k\in[0,\ldots,23]}$	Hour frequencies of orders recorded over week days
8	$(d^{-1}([5,6]) \cap h^{-1}(k) \times \mathcal{X})_{k\in[0,\ldots,23]}$	Hour frequencies of orders recorded over weekends
9	$(h^{-1}(k) \times \mathcal{X})_{k\in[0,\ldots,23]}$	Hour frequencies of orders recorded overall
10	$(d^{-1}([0,\ldots,4]) \times \mathcal{P})$	Number of products ordered over week days
11	$(d^{-1}([5,6]) \times \mathcal{P})$	Number of products ordered over weekends
12	$(\mathcal{T} \times \mathcal{P})$	Number of products ordered overall

size of the data collection. For the location dataset, M is the number of week in the time period $\mathcal{T}$, e.g. $M_i = M_{\text{aux}} = 5$ for Θ_{aux} and $M_i = M_{\text{data}} = 10$ for Θ_{data}. For the grocery shopping dataset, $M_i = \#y_i$ is the number of orders placed by a person, i.e. $80\% \times M_{\text{aux}} = 20\% \times M_{\text{data}}$ due to the 80/20 split for Θ_{test} and Θ_{aux}. Overall:

$$Z_{i,k} = \begin{cases} \frac{N_i(B)}{M_i} & \text{if } \mathcal{B}_k = \{B\} \\ \left(\frac{N_i(B)}{\sum\limits_{B'\in\mathcal{B}_k} N_i(B')}\right)_{B\in\mathcal{B}_k} & \text{otherwise.} \end{cases} \tag{S1}$$

Profiles are compared using the model divergence $d_{\Omega,\Lambda}$. Considering the previous paragraph, for q countable collections $\mathcal{B}_1, \ldots, \mathcal{B}_q$ of Borel sets with $\mathcal{C} = \{k \mid \#\mathcal{B}_k = 1\}$ and $c = \#\mathcal{C}$, the set of profiles is $\mathcal{S} = \prod\limits_{k=1,\ldots,q-c} \mathcal{S}_k \times \mathbb{R}_+^c$. The divergence $d_{\Omega,\Lambda} = \sum\limits_{k=1}^{q} \Omega_k \, d_{\Lambda_k}$ is defined for parameters $\Omega \in \mathbb{R}_+^q$ and $\Lambda \in (0,1)^q$. For indexes $k \in \mathcal{C}$, Λ_k is fixed and unused ($\Lambda_k = \frac{1}{2}$ for instance) and the sub-divergence d_{Λ_k} is chosen as the relative difference, i.e. $d_{\Lambda_k}(x,y) = \frac{|x-y|}{x+y}$ for any $x, y \in \mathbb{R}_+$, with Ω_k acting as a normalization factor. For indexes $k \notin \mathcal{C}$, according to the formula given in the main text, the sub-divergence $d_{\Lambda_k}(X_k \parallel Y_k)$ is the mutual information between a Bernoulli variable B of parameter Λ_k independent from X_k and Y_k, and a random variable obtained by mixing X_k and Y_k with B, i.e. $BX_k + (1-B)Y_k$.

S2.2 Gradient Descent

The divergence $d_{\Omega,\Lambda}$ is continuously differentiable with respect to parameters Ω and Λ. For all $\Psi_i = (Z_{i,k})_{k=1,\ldots,q}$ and $\Psi_{i'} = (Z_{i',k})_{k=1,\ldots,q} \in \mathcal{S}$ and $1 \le k \le q$:

$$\begin{cases} \frac{\partial d_{\Omega,\Lambda}}{\partial \Omega_k}(\Psi_i, \Psi_{i'}) = d_{\Lambda_k}(Z_{i,k} \| Z_{i',k}) \\ \frac{\partial d_{\Omega,\Lambda}}{\partial \Lambda_k}(\Psi_i, \Psi_{i'}) = 1_{\{k\in\mathcal{C}\}} \left[\mathrm{KL}(Z_{i',k} \parallel M_{i,i',k}) - \mathrm{KL}(Z_{i,k} \parallel M_{i,i',k})\right] \end{cases} \tag{S2}$$

where $M_{i,i',k} = \Lambda_k Z_{i,k} + (1-\Lambda_k) Z_{i',k} \in \mathcal{S}_k$ by convex combination. Eq. S2 shows

that partial derivatives $\frac{\partial d_{\Omega,\Lambda}}{\partial \Omega_k}$ quantify how each collection $\mathcal{B}_k$ of Borel sets discriminate between two profiles. Furthermore, partial derivatives $\frac{\partial d_{\Omega,\Lambda}}{\partial \Lambda_k}$ quantify discrepancies on the probability simplex $\mathcal{S}_k$ with respect to the Kullback-Leibler divergence. In particular, for $k \in \mathcal{C}$, $\frac{\partial d_{\Omega,\Lambda}}{\partial \Lambda_k} = 0$ if and only if $\mathrm{KL}(Z_{i',k} \parallel M_{i,i',k}) = \mathrm{KL}(Z_{i,k} \parallel M_{i,i',k})$. With these expressions, gradients can be computed explicitly.

The loss function is minimized by an Adam gradient descent algorithm [96], using standard meta-parameters (step-size $\gamma = 0.001$, $\beta_1 = 0.9$, $\beta_2 = 0.999$, small constant $\mu = 10^{-8}$). At each step t, using the current values Ω_t, Λ_t, traces in Θ_1 are compared to all traces in Θ_2. This yields two training subsets $\Theta_{1,t,+} = \{y_1 \in \Theta_1 \mid \arg\min_{y \in \Theta_2} d_{\Omega_t,\Lambda_t}(\Psi(y_1) \parallel \Psi(y_2)) \equiv y_1\}$ (positive examples) and $\Theta_{1,t,-} = \{y_1 \in \Theta_1 \mid \arg\min_{y \in \Theta_2} d_{\Omega_t,\Lambda_t}(\Psi(y_1) \parallel \Psi(y_2)) \not\equiv y_1\}$ (negative examples).

To speed up the training, a candidate list $\ell_{\tau,t,\phi}$ containing the closest ψ traces to τ for the current divergence d_{Ω_t,Λ_t} is stored in memory for $\phi = 50$ iterations and used, instead of the whole set of training traces Θ_2, when computing $\Theta_{1,t,+}$ and $\Theta_{1,t,-}$, i.e. $\hat{\Theta}_{1,t,+} = \{y_1 \in \Theta_1 \mid \arg\min_{y \in \ell_{y_1,t,\phi}} d_{\Omega_t,\Lambda_t}(\Psi(y_1) \parallel \Psi(y_2)) \equiv y_1\}$ and $\hat{\Theta}_{1,t,-} = \{y_1 \in \Theta_1 \mid \arg\min_{y \in \ell_{y_1,t,\phi}} d_{\Omega_t,\Lambda_t}(\Psi(y_1) \parallel \Psi(y_2)) \not\equiv y_1\}$. Every ϕ iterations, candidates lists $\ell_{\tau,t,\phi}$ are updated by using Θ_1 and Θ_2 again. Over the whole training procedure ($\mathcal{M} = 2000$ iterations), this method reduces the total complexity for obtaining information about positive and negative examples from $O(\mathcal{M}\#\Theta_1\#\Theta_2)$ to $O(\mathcal{M}\#\Theta_1(\frac{\#\Theta_2}{\phi} + h))$. For the location dataset, we use a training size of $10,000$ individuals and $\psi = 100$ candidates. For the grocery shopping dataset, we use a training size of $5,000$ individuals and $\psi = 200$ candidates. Training sizes were kept small to maximize the amount of individuals available for testing (see Fig. S1). The values of ϕ and ψ were selected empirically, with discrepancies reflecting that identification is harder on the grocery shopping dataset.

Gradients are computed explicitly by averaging the expressions in Eq. S2 on a mini batch θ_t of 200 individuals: 100 individuals from $\hat{\Theta}_{1,t,+}$ and 100 $\hat{\Theta}_{1,t,-}$, all sampled uniformly at each step t. Early stopping is used after the training curve flattens, or otherwise after $\mathcal{M} = 2000$ iterations. The step t^* with the highest validation accuracy is selected, and $\Omega(\alpha) = \Omega_{t^*}$ and $\Lambda(\alpha) = \Lambda_{t^*}$. We finally perform a grid search over the parameter α to maximise the validation accuracy $\zeta_1^{\mathrm{Valid}}(\alpha)$, and obtain $\alpha^* = \arg\max_{\alpha>0} \zeta_1^{\mathrm{Valid}}(\alpha)$. Parameters of the model at the end of training are $\Omega = \Omega(\alpha^*)$ and $\Lambda = \Lambda(\alpha^*)$.

Identification performances are monitored by computing the training accuracy from traces in Θ_1 against Θ_2 and the validation accuracy from traces in Θ_3 against Θ_4 at each step t of the gradient descent with current values Ω_t, Λ_t. Importantly, these accuracies cannot be compared to one another. Indeed, identification performances depend on the number of individuals in the dataset ($10,000$ in training, $1,000$ in validation, $500,000$ in testing for the location dataset), as shown in the main text, as well as on the temporal length of the splits used for training, validation, and testing. For instance, for the loca-

tion dataset, Θ_1 and Θ_2 are each 4-week long, Θ_3 and Θ_4 are each 1-week long, Θ_{data} is 10-week long, and Θ_{aux} is 5-week long (see Fig. S1). Relative improvements in both training and validation accuracies ζ_1^{Train} and ζ_1^{Valid}, however, indicate that the model better identifies disjoint sets of individuals over disjoint periods of time, suggesting that testing performances should also improve.

Fig. S2 (resp. Fig. S3) shows how training accuracy ζ_1^{Train} and validation accuracy ζ_1^{Valid} evolve during training for the location (resp. grocery shopping) dataset. We observe consistent improvement across training and validation, as ζ_1^{Train} and ζ_1^{Valid} both peak after approximately the same number of steps, indicating that training works well. We further observe that the value of the meta parameter α strongly influences the evolution of both ζ_1^{Train} and ζ_1^{Valid}. The optimal value α^* for a given dataset is found by the attacker by grid-search. We observe α^* getting smaller as the unknown amount of noise added to the dataset is increased and the identification becomes more challenging. This suggests that when noise is added to the dataset the values of $\mathcal{D}(\theta_+)$ increase, requiring the balance between $\mathcal{D}(\theta_-)$ and $\mathcal{D}(\theta_+)$ to be slightly adjusted to continue to train the model effectively.

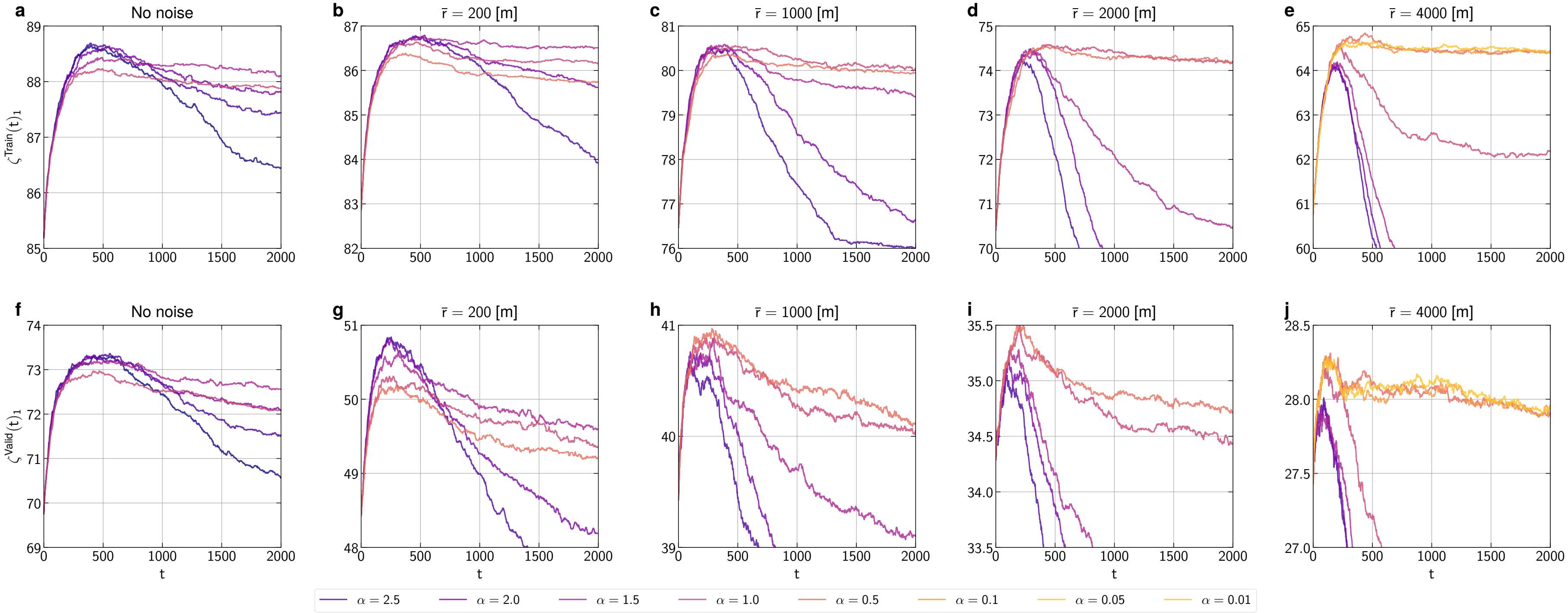

Figure S2: Training (top row) and validation (bottom row) accuracies during the training phase for increasing amounts of added noise (location dataset). Importantly, as discussed in the text, ζ_1^{Train}, ζ_1^{Valid}, and ζ_1^{Test} cannot be compared to one another, as identification accuracy depends on the sizes of both the individual and the temporal splits. Accuracies ζ_1^{Train} and ζ_1^{Valid} increase noticeably in all cases over the first steps for all values of α. ζ_1^{Train} and ζ_1^{Valid} peak approximately after the same number of steps indicating that training works. For the optimal value α^*, training accuracy barely decreases after hitting the peak whereas the validation accuracy decreases slightly. This is a common phenomenon suggesting that the model might be starting to overfit the divergence for the individuals in the training set and that training should be stopped (early stopping criterion). For instance, without noise, the selected model was $\alpha^* = 1.4$ at step $t^* = 441$.

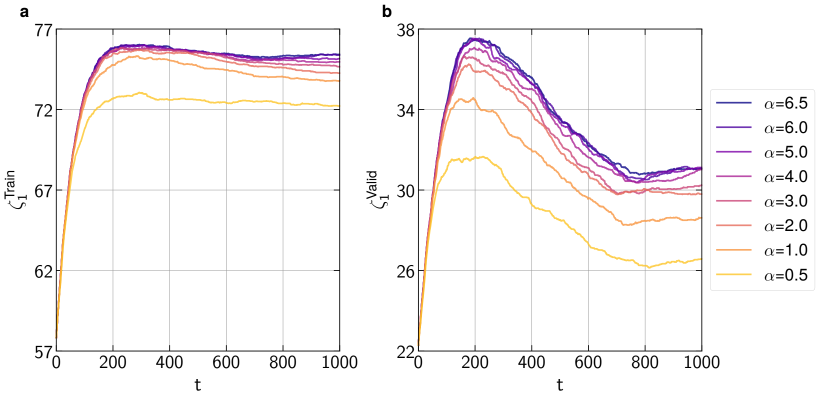

Figure S3: Training (a) and validation (b) accuracies during the training phase (grocery shopping dataset). Accuracies ζ_1^{Train} and ζ_1^{Valid} increase noticeably over the first 100 steps for all values α. After peaking between $t = 100$ and $t = 300$ steps, validation accuracy decreases. The selected model was $\alpha^* = 6.2$ at step $t^* = 199$. ζ_1^{Train}, ζ_1^{Valid}, and ζ_1^{Test} cannot be compared to one another.

S2.3 t-SNE visualisation

We select, for the t-SNE plot, one individual incorrectly identified by our model before training but correctly identified after training, along with the 4 people who are the most similar to this individual after training. This allows us to visualise how the training improves the model ability to identify this individual, and distinguish them from similar individuals. We note $\Theta_{\text{TSNE}} = \{\tau_\mu^\eta \mid 1 \leq \eta \leq u, 1 \leq \mu \leq v\}$ the traces obtained from each single week η from the $u = 15$ consecutive weeks in $\mathcal{T}_{\text{data}} \cup \mathcal{T}_{\text{aux}}$ for these $v = 5$ individuals re-indexed. The matrix of divergences Δ between each of the resulting traces is then computed and the t-SNE algorithm [97] is applied to Δ. Formally, for % the modulo operator and / the Euclidian division:

$$\forall \quad 0 \leq k, \ell < uv \quad \Delta_{k,\ell} = d_{\Omega,\Lambda}(\Psi(\tau_{1+k/u}^{1+k\%u}), \Psi(\tau_{1+\ell/u}^{1+\ell\%u})) \tag{S3}$$

Conditional probabilities $p_{k|\ell}$ are computed with:

$$p_{k|\ell} = \frac{\exp(-\Delta_{k,\ell}/2\sigma_k^2)}{\sum\limits_{m \neq k} \exp(-\Delta_{k,m}/2\sigma_k^2)} \tag{S4}$$

From these conditional probabilities, we define the symmetric joint distribution matrix $P_{k,\ell} = \frac{p_{k|\ell}+p_{\ell|k}}{2uv}$ used by the algorithm to represent how profiles are distributed in the input space, i.e. the space of profiles $\mathcal{S}$ here.

To obtain a bi-dimensional representation of the profiles, points $z_k \in \mathbb{R}^2$ are computed to follow the symmetric joint distribution matrix $Q_{k,\ell} = \frac{(1+\|z_k - z_\ell\|)^{-1}}{\sum\limits_{m \neq k} (1+\|z_k - z_m\|)^{-1}}$ where $\|.\|$ is the Euclidean distance in $\mathbb{R}^2$. More specifically, the Kullback-Leibler divergence $\text{KL}(P \parallel Q) = \sum\limits_{k \neq \ell} p_{k,\ell} \log(\frac{p_{k,\ell}}{q_{k,\ell}})$ between the distribution matrix in the profile space $\mathcal{S}$ and the distribution matrix in the Cartesian plane $\mathbb{R}^2$ is minimized by gradient descent. The confidence ellipses

Model	Scenario 1	Scenario 2	Scenario 3
Markov-based [71]	26.5%	23.9%	16.3%
Histogram (Cosine)	51.4%	46.9%	17.9%
Histogram (L1)	60.9%	53.3%	16.8%
Histogram (Bhat)	65.1%	59.3%	31.8%
Histogram (JS)	65.2%	59.6%	30.3%
Profiling (ours)	**78.8%**	**75.9%**	**58.7%**

Table S3: Our profiling attack strongly outperforms the state-of-the-art. We consider different scenarios with and without noise added to the dataset (scenario 1 no noise, scenario 2 small average noise $\bar{r} = 200$m, and scenario 3 very large average noise $\bar{r} = 2000$m). We find that our model outperforms all Markov-based and histogram-based attacks across all scenarios. Other Markov-based attacks, e.g. [72] were below 10% accuracy in the easiest scenario 1.

drawn around the resulting points are 2σ confidence ellipses.

S3 Supplementary Results

S3.1 Comparison with previous works

In this paper, we have considered a general attack where the auxiliary information of a single individual is compared to a dataset of N individuals. Some of the previous studies had instead considered a simpler approach, known as N-to-N assignment, where the auxiliary information and the dataset form a complete bipartite graph, i.e. $I_{\text{aux}} = I_{\text{data}}$. This simplifies the problem to searching for a permutation of identities between all these auxiliary information and the dataset, usually using the Hungarian algorithm [99]. This however relies on the strong and often unrealistic assumption that the attacker has, as auxiliary information, a complete auxiliary dataset with information about exactly every single person in the original dataset, and only them. We therefore compare previous work with ours without this assumption.

Table S3 compares our method to previous work across our three scenarios for $N = 500,000$ individuals. Our method strongly outperforms all Markov-based and histogram-based methods from the literature in all three scenarios.

S3.1.1 Markov-based methods

The first class of profiling attacks studied in the literature revolves around the assumption of Markov dependencies between records. In the case of location data, many studies have proposed and investigated Mobility Markov Chains [71, 72, 100]. For sparse data, e.g. the CDR dataset used in this paper, the method proposed by Gambs et al. [71] is the state of the art.

We here summarize this method. Assuming that the k most visited locations of an individual (their "points of interest") represent the states a Markov Chain characterising the mobility of this individual, transition probabilities are extracted from the traces from

Θ_{data} and Θ_{aux}. For $\tau \in \Theta_{\text{aux}}$ and $y \in \Theta_{\text{data}}$, we note V_τ and V_y their stationary vectors computed for the k points of interests P_τ and P_y, each ranked in decreasing frequency of visit order. The dissimilarity function used to compare individuals is a combination of two distances: the stationary distance, measuring the geographical distance between the locations of the points of interests, and the proximal distance, measuring discrepancies between these point of interests. If the stationary distance is above a given threshold s, determined on the training sets Θ_1 and Θ_2, the proximity distance is used instead:

$$D_{\text{Gambs},s}(\tau, y) = d_{\text{stationary}}(\tau, y) 1_{d_{\text{stationary}}(\tau,y) \leq s} + d_{\text{proximal}}(\tau, y, r, \Delta) 1_{d_{\text{stationary}}(\tau,y) > s} \quad \text{(S5)}$$

For $\text{mdv}(P, P') = (\min_{x' \in P'} \|x - x'\|)_{x \in P}$ the minimum distance vector between the geographical locations of P and P' for the Euclidean distance $\|.\|$

$$d_{\text{stationary}}(\tau, y) = \frac{V_\tau \cdot \text{mdv}(P_\tau, P_y) + V_y \cdot \text{mdv}(P_y, P_\tau)}{2} \quad \text{(S6)}$$

Using parameters recommended by the authors [71], i.e. a distance threshold $\Delta = 5$km, we note $c_\Delta(P, P') = (1_{\|x_i - x'_i\| < \Delta})_{i=0,\ldots,\min(\#P,\#P')}$ the proximity (Boolean) vector, and for a parameter $r = 10$, the weight vector $w_r(P, P') = (\max(1, \frac{r}{2^i}))_{i=0,\ldots,\min(\#P,\#P')}$. The proximal distance is defined by

$$d_{\text{proximal}}(\tau, y, r, \Delta) = \frac{1}{c_\Delta(P, P') \cdot w_r(P_\tau, P_y)} \quad \text{(S7)}$$

The maximum number of point of interests k to use per individual is then determined by grid search using the training sets Θ_1 and Θ_2, for $k \in [4, 8]$.

S3.1.2 Histogram-based methods

The other class of profiling attacks researched in the literature revolves around the comparison of histograms. Our profiling attack is more closely related to these approaches.

The most notorious method, proposed by Naini et al. [73], computes the histogram of visited locations for each individual in the dataset, and compares it with the histogram of the auxiliary information using the Jensen-Shannon divergence. With the JS notation for the Jensen-Shannon divergence, and $\Gamma(\tau)$ the location histogram of a trace τ over the recording period, the dissimilarity function used by the algorithm is:

$$D_{\text{Naini}}(\tau, y) = \text{JS}(\Gamma(\tau), \Gamma(y)) \quad \text{(S8)}$$

However, as pointed out by the authors, many other divergences can and have also been used, including the widely used cosine divergence [76], L1 distance [75], and Bhattacharyya distance [74].

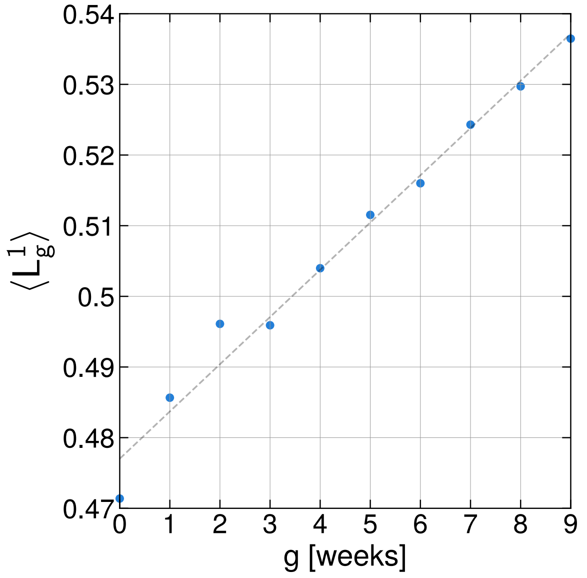

Figure S4: Human behavior drift over time. Evolution of the average L^1 distance between the location histogram of an individual in the dataset compared to the same individual over $\mathcal{T}_{\text{aux},g}$, as the time gap g increases. Values ranges from 0 (identical) to 2 and are averaged over the 420,787 individuals considered in the time gap experiment (linear fit, $R^2 = 0.98$).

S3.2 Robustness of the model over time

We showed in the main text the robustness of our model to behavioral changes over time (Fig. S4) by shifting the time period $\mathcal{T}_{\text{aux}}$ corresponding to the auxiliary information to $\mathcal{T}_{\text{aux},g}$ (for $g = 0, \ldots, 9$) (Fig. S5). The same model was used for all values of g, meaning that the model was trained once and applied independently to all $\mathcal{T}_{\text{aux},g}$ without retraining. This is due to the training and validation procedures being done on Θ_1, Θ_2, Θ_3, and Θ_4, which are all recorded on sub-intervals of the period $\mathcal{T}_{\text{data}}$ and left unchanged as the gap g increased.

S3.3 Determinants of identifiability

We studied a handful of summary statistics in the main text to understand why some individuals might be easier to identify than others. This analysis was performed a posteriori using the Kruskal-Wallis test [96], a nonparametric analogous to the one way analysis of variance (ANOVA) that does not assume normal distributions of the summary statistics and compares instead the medians of each group. Other tests could have been chosen, such as the nonparametric Kolmogorov-Smirnov test [101]. The KS-test, however, leads to the same conclusion, albeit with slightly different p-values.

Fig. S6 shows the cumulative distribution functions (CDF) of these summary statistics computed over Θ_{data} for individuals in group 1 and group 2. The radius of gyration [56] of an individual i is defined as $R_i = \sqrt{\frac{1}{n_i^u} \sum_{j=1}^{n_i^u} \|\mathbf{x}_{j,i} - \langle \mathbf{x} \rangle_i \|^2}$ where $\|.\|$ is the Euclidean

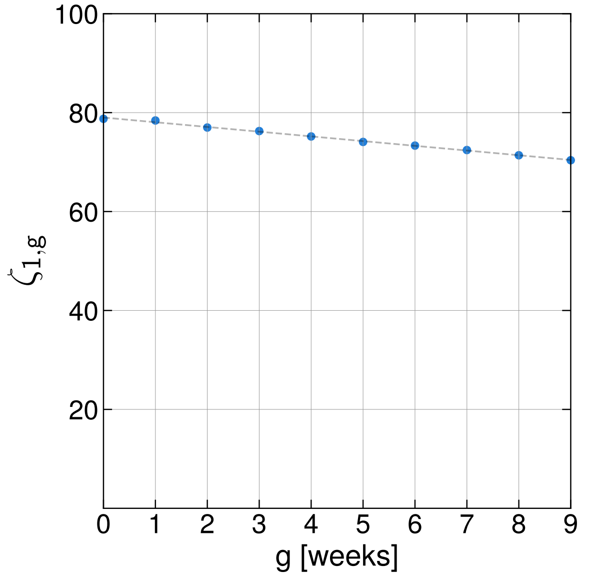

Figure S5: The model accuracy slowly decreases over time. Evolution of $\zeta_{1,g}$ as the time gap g between the end of $\mathcal{T}_{\text{data}}$ and the beginning of $\mathcal{T}_{\text{aux}}$ increases. The average drop of $\zeta_{1,g}$ is 1% per added week (±0.3, SED).

distance between geographical positions, $\langle \mathbf{x} \rangle_i = \frac{1}{n_i^u} \sum_{j=1}^{n_i^u} \mathbf{x}_{j,i}$ the uniform center of mass of the trajectory, and n_i^u the number of unique antenna cells visited by individual i. The entropy is obtained using the overall location distribution, computed using the collection of Borel sets $\mathcal{B}_3$ (see Table S1). The area of a cell is computed using the so-called Surveyor's area formula [102] from the Euclidean Voronoi tessellation [103].

S4 Supplementary Tables

Threshold κ	Score Threshold s^κ	False Discovery Rate
90%	0.1384	9.61%
95%	0.2313	4.81%
99%	0.5009	0.91%

Table S4: False Discovery Rates obtained by the meta-classifier on the location dataset for various thresholds. For each threshold κ, we report both the corresponding score threshold value s^κ and the resulting FDR.

Threshold κ	Score Threshold s^κ	False Discovery Rate
90%	0.1493	8.90%
95%	0.2467	4.22%
99%	0.5887	0.72%

Table S5: False Discovery Rates obtained by the meta-classifier on the grocery shopping dataset for various thresholds. For each threshold κ, we report both the corresponding score threshold value s^κ and the resulting FDR.

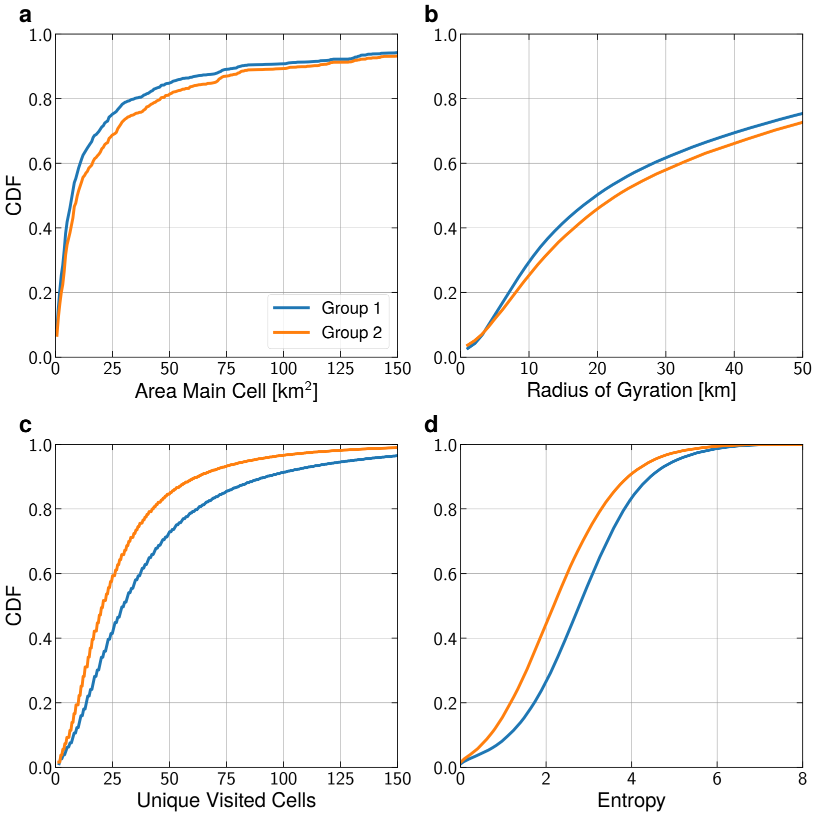

Figure S6: Post-hoc analysis of summary statistics. Empirical Cumulative Distribution Function (CDF) computed over the individuals in each group for **a.** the area of the main visited cell, **b.** the radius of gyration, **c.** the number of unique visited cells, and **d.** the entropy of the location distribution over the antenna cells. Individuals that are more identifiable (group 1) visit more unique locations (30 vs 21 medians, p-value $p < 10^{-15}$), their traces contains more geographical information (geographical entropy of traces - 2.8 vs 2.2 bits of information, $p < 10^{-15}$), they spend most of their time within a small geographical region (radius of gyrations [56] - 19.8 vs 21.8 km, $p < 10^{-15}$), and they live in less densely populated area (area of the primary Voronoi cell - 7.2 vs 9.6 km^2, $p < 10^{-15}$) than individuals in group 2.

Prior p	Score Threshold $s^{\kappa=95\%}$	False Discovery Rate
50%	0.7910	3.86%
75%	0.4649	4.44%
90%	0.3182	4.74%

Table S6: False Discovery Rates obtained by the meta-classifier on the location dataset for various prior. For each prior p, we report both the corresponding score threshold value $s^{\kappa=95\%}$ and the resulting FDR.

Prior p	Score Threshold $s^{\kappa=95\%}$	False Discovery Rate
50%	0.4363	4.27%
75%	0.4469	4.06%
90%	0.4380	4.22%

Table S7: False Discovery Rates obtained by the meta-classifier on the grocery shopping dataset for various prior. For each prior p, we report both the corresponding score threshold value $s^{\kappa=95\%}$ and the resulting FDR. Contrary to the location dataset, for which a large number of individuals were still available beyond the 0.5M used in the main text, results here were obtained by sampling a smaller dataset ($\#\Theta_A = \#\Theta_B = 40,000$ individuals) to simulate targets not contained in the dataset.

N	Profiling (Ours)	Histogram (JS)	Histogram (Bhat)	Histogram (L1)	Histogram (Cosine)
1	**100%**	**100%**	**100%**	**100%**	**100%**
10	**100%**	**100%**	**100%**	**100%**	**100%**
25	**100%**	**100%**	**100%**	**100%**	**100%**
50	**100%**	**100%**	**100%**	**100%**	99.80%(±0.37)
100	**99.90**%(±0.19)	99.80%(±0.25)	99.80%(±0.25)	99.80%(±0.25)	99.30%(±0.40)
250	**99.72**%(±0.16)	99.44%(±0.30)	99.52%(±0.22)	99.00%(±0.30)	98.40%(±0.53)
1000	**98.86**%(±0.14)	97.57%(±0.27)	97.67%(±0.27)	97.11%(±0.32)	94.60%(±0.43)
2500	**97.85**%(±0.15)	95.49%(±0.28)	95.54%(±0.28)	94.40%(±0.40)	90.18%(±0.22)
5000	**96.67**%(±0.09)	93.25%(±0.24)	93.19%(±0.21)	91.77%(±0.25)	86.26%(±0.17)
10000	**95.17**%(±0.16)	90.11%(±0.14)	90.02%(±0.13)	88.28%(±0.21)	81.55%(±0.24)
25000	**92.64**%(±0.16)	85.01%(±0.10)	84.89%(±0.13)	82.72%(±0.12)	74.69%(±0.15)
50000	**90.18**%(±0.08)	80.60%(±0.10)	80.46%(±0.08)	77.96%(±0.11)	69.27%(±0.13)
100000	**87.13**%(±0.09)	75.97%(±0.06)	75.85%(±0.07)	72.89%(±0.07)	64.82%(±0.05)
150000	**85.15**%(±0.10)	73.33%(±0.10)	73.16%(±0.10)	69.86%(±0.13)	60.50%(±0.09)
200000	**83.92**%(±0.11)	71.37%(±0.10)	71.24%(±0.10)	67.80%(±0.09)	58.44%(±0.08)
250000	**82.60**%(±0.07)	69.86%(±0.11)	69.65%(±0.11)	66.24%(±0.13)	56.78%(±0.09)
300000	**81.55**%(±0.07)	68.53%(±0.12)	68.35%(±0.13)	64.75%(±0.09)	55.16%(±0.08)
350000	**80.72**%(±0.12)	67.52%(±0.10)	67.33%(±0.09)	63.62%(±0.11)	54.02%(±0.09)
400000	**80.10**%(±0.12)	66.68%(±0.10)	66.46%(±0.09)	62.81%(±0.10)	53.15%(±0.11)
450000	**79.31**%(±0.11)	65.86%(±0.07)	65.64%(±0.10)	61.78%(±0.09)	52.13%(±0.09)
500000	**78.75%**	65.20%	65.09%	60.97%	51.36%

Table S8: Accuracies in the scalability experiment for the location dataset. Each value is reported after averaging over 10 runs, with reported errors corresponding to 95% confidence intervals. No errors are reported when either the 10 values obtained were equal (100%) or when no randomization was possible (the entire test set is used).

N	Accuracy
1	100%
10	100%
25	96.44%(±1.93)
50	94.22%(±1.44)
100	90.78%(±1.18)
150	88.07%(±1.37)
200	87.05%(±1.57)
250	85.38%(±1.93)
500	82.82%(±1.01)
1000	80.10%(±0.99)
5000	74.28%(±0.35)
10000	72.11%(±0.16)
15000	70.74%(±0.21)
20000	69.84%(±0.11)
25000	69.13%(±0.06)
30000	68.52%(±0.08)
35000	68.02%(±0.05)
40000	67.59%(±0.05)
45000	67.22%(±0.07)
50000	66.90%(±0.07)
55000	66.63%(±0.06)
60000	66.34%(±0.05)
65000	66.12%(±0.03)
70000	65.88%(±0.03)
75000	65.66%(±0.05)
80000	65.44%(±0.04)
85000	65.26%(±0.01)

Table S9: Accuracies in the scalability experiment for the grocery shopping dataset. Each value is reported after averaging over 10 runs, with reported errors corresponding to 95% confidence intervals (no errors are reported when the 10 values obtained were equal (100%)).

REFERENCES AND NOTES

1. Strategy Analytics, “Global connected and IoT device forecast update” (Strategy Analytics, 2019).

2. J. Valentino-Devries, N. Singer, M. H. Keller, A. Krolik, “Your apps know where you were last night, and they’re not keeping it secret,” *New York Times*, 10 December 2018; www.nytimes.com/interactive/2018/12/10/bsiness/location-data-privacy-apps.html [accessed 4 January 2021].

3. Communication: A European strategy for data; https://ec.europa.eu/info/strategy/priorities-2019-2024/europe-fit-digital-age/european-data-strategy [accessed 11 December 2020].

4. P. Burge, J. Shawe-Taylor, An unsupervised neural network approach to profiling the behavior of mobile phone users for use in fraud detection. *J. Parallel Distrib. Comput.* **61**, 915–925 (2001).

5. D. Björkegren, D. Grissen, Behavior revealed in mobile phone usage predicts credit repayment. *World Bank Econ. Rev.* **34**, 618–634 (2020).

6. J. L. Toole, Y.-R. Lin, E. Muehlegger, D. Shoag, M. C. González, D. Lazer, Tracking employment shocks using mobile phone data. *J. R. Soc. Interface* **12**, 20150185 (2015).

7. L. Bengtsson, X. Lu, A. Thorson, R. Garfield, J. V. Schreeb, Improved response to disasters and outbreaks by tracking population movements with mobile phone network data: A post-earthquake geospatial study in Haiti. *PLOS Med.* **8**, e1001083 (2011).

8. T. Breton, Commission recommendation (EU) 2020/518 of 8 April 2020 on a common union toolbox for the use of technology and data to combat and exit from the COVID-19 crisis, in particular concerning mobile applications and the use of anonymised mobility data (2020); https://eur-lex.europa.eu/eli/reco/2020/518/oj [accessed 23 February 2021].

9. K. H. Grantz, H. R. Meredith, D. A. T. Cummings, C. J. E. Metcalf, B. T. Grenfell, J. R. Giles, S. Mehta, S. Solomon, A. Labrique, N. Kishore, C. O. Buckee, A. Wesolowski, The use of

mobile phone data to inform analysis of COVID-19 pandemic epidemiology. *Nat. Commun.* **11**, 4961 (2020).

10. N. Oliver, B. Lepri, H. Sterly, R. Lambiotte, S. Deletaille, M. De Nadai, E. Letouzé, A. A. Salah, R. Benjamins, C. Cattuto, V. Colizza, N. de Cordes, S. P. Fraiberger, T. Koebe, S. Lehmann, J. Murillo, A. Pentland, P. N. Pham, F. Pivetta, J. Saramäki, S. V. Scarpino, M. Tizzoni, S. Verhulst, P. Vinck, Mobile phone data for informing public health actions across the COVID-19 pandemic life cycle. *Sci. Adv.* **6**, eabc0764 (2020).

11. A. Wesolowski, N. Eagle, A. J. Tatem, D. L. Smith, A. M. Noor, R. W. Snow, C. O. Buckee, Quantifying the impact of human mobility on malaria. *Science* **338**, 267–270 (2012).

12. X. Dong, A. J. Morales, E. Jahani, E. Moro, B. Lepri, B. Bozkaya, C. Sarraute, Y. Bar-Yam, A. Pentland, Segregated interactions in urban and online space. *EPJ Data Sci.* **9**, 20 (2020).

13. E. F. Stone, D. L. Stone, Privacy in organizations: Theoretical issues, research findings, and protection mechanisms. *Res. Pers. Hum. Resour. Manag.* **8**, 349–411 (1990).

14. K. Granville, “Facebook and Cambridge Analytica: What you need to know as fallout widens,” *New York Times*, 19 March 2018; www.nytimes.com/2018/03/19/technology/facebook-cambridge-analytica-explained.html.

15. D. Lyon, Surveillance, Snowden, and big data: Capacities, consequences, critique. *Big Data Soc.* **1**, 2053951714541861 (2014).

16. Morning Consult, National tracking poll #210496 (2021); https://assets.morningconsult.com/wp-uploads/2021/04/26163900/210496_crosstabs_MC_TECH_RVs_v1_LM.pdf

17. Harris Interactive, Information rights strategic plan: Trust and confidence (2019); https://ico.org.uk/media/about-the-ico/documents/2615515/ico-trust-and-confidence-report-20190626.pdf.

18. Recital 26: Not applicable to anonymous data (2018); https://gdpr.eu/recital-26-not-applicable-to-anonymous-data/ [accessed 6 December 2020].

19. California State Legislature, California consumer privacy act of 2018 (2018); https://www.consumerprivacyact.com/section-1798-140-definitions/.

20. Concerning the management, oversight, and use of data; https://app.leg.wa.gov/billsummary?BillNumber=5062&Year=2021&Initiative=false [accessed 24 February 2021].

21. An act relative to data privacy; https://malegislature.gov/Bills/192/HD3847 [accessed 24 February 2021].

22. Consumer data protection act; https://lis.virginia.gov/cgi-bin/legp604.exe?211+ful+HB2307H1 [accessed 24 February 2021].

23. H. L. Dunn, Record linkage. *Am. J. Public Health* **36**, 1412–1416 (1946).

24. L. Sweeney, Computational disclosure control for medical microdata: The Datafly system, in *Record Linkage Techniques 1997: Proceedings of an International Workshop and Exposition* (National Academy Press, 1997), pp. 442–453.

25. B. Malin, L. Sweeney, Re-identification of DNA through an automated linkage process, in *Proceedings of the AMIA Symposium* (American Medical Informatics Association, 2001), p. 423.

26. P. Ohm, Broken promises of privacy: Responding to the surprising failure of anonymization. *UCLA Law Rev.* **57**, 1701 (2009).

27. R. Buettner, S. Craig, "Decade in the red: Trump tax figures show over $1 billion in business losses," *New York Times*, 8 May 2019, p. 7.

28. L. Sweeney, k-anonymity: A model for protecting privacy. *Int. J. Uncertain. Fuzziness Knowl.-Based Syst.* **10**, 557–570 (2002).

29. G. J. Matthews, O. Harel, Data confidentiality: A review of methods for statistical disclosure limitation and methods for assessing privacy. *Statist. Surv.* **5**, 1–29 (2011).

30. C. Skinner, Statistical disclosure control for survey data, in *Handbook of Statistics* (Elsevier, 2009), vol. 29, pp. 381–396.

31. D. Kifer, Attacks on privacy and Definetti's theorem, in *Proceedings of the 2009 ACM SIGMOD International Conference on Management of data* (Association for Computing Machinery, 2009), pp. 127–138.

32. R. Kumar, J. Novak, B. Pang, A. Tomkins, On anonymizing query logs via token-based hashing, in *Proceedings of the 16th International Conference on World Wide Web* (Association for Computing Machinery, 2007), pp. 629–638.

33. A. Lavrenovs, K. Podins, Privacy violations in Riga open data public transport system, in *2016 IEEE 4th Workshop on Advances in Information, Electronic and Electrical Engineering (AIEEE)* (IEEE, 2016), pp. 1–6.

34. Y.-A. de Montjoye, L. Radaelli, V. K. Singh, A. S. Pentland, Unique in the shopping mall: On the reidentifiability of credit card metadata. *Science* **347**, 536–539 (2015).

35. A. D. Luzio, A. Mei, J. Stefa, Consensus robustness and transaction de-anonymization in the ripple currency exchange system, in *2017 IEEE 37th International Conference on Distributed Computing Systems (ICDCS)* (IEEE, 2017), pp. 140–150.

36. R. Pellungrini, L. Pappalardo, F. Pratesi, A. Monreale, A data mining approach to assess privacy risk in human mobility data. *ACM Trans. Intell. Syst. Technol.* **9**, 1–27 (2017).

37. Y.-A. de Montjoye, C. A. Hidalgo, M. Verleysen, V. D. Blondel, Unique in the crowd: The privacy bounds of human mobility. *Sci. Rep.* **3**, 1376 (2013).

38. V. Sekara, E. Mones, H. Jonsson, Temporal limits of privacy in human behavior. arXiv:1806.03615 [cs.CY] (10 June 2018).

39. J. Su, A. Shukla, S. Goel, A. Narayanan, De-anonymizing web browsing data with social networks, in *Proceedings of the 26th International Conference on World Wide Web* (International World Wide Web Conferences Steering Committee, 2017), pp. 1261–1269.

40. C. Deußer, S. Passmann, T. Strufe, Browsing unicity: On the limits of anonymizing web tracking data, in *2020 IEEE Symposium on Security and Privacy (SP)* (IEEE, 2020), pp. 777–790.

41. A. Narayanan, V. Shmatikov, Robust de-anonymization of large sparse datasets, in *Proceedings of the 2008 IEEE Symposium on Security and Privacy* (IEEE, 2008), pp. 111–125.

42. S. Cleemput, M. A. Mustafa, E. Marin, B. Preneel, De-pseudonymization of smart metering data: Analysis and countermeasures, in *2018 Global Internet of Things Summit (GIoTS)* (IEEE, 2018), pp. 1–6.

43. M. Hay, G. Miklau, D. Jensen, P. Weis, S. Srivastava, Anonymizing social networks, in *Computer Science Department Faculty Publication Series* (2007), p. 180.

44. A. Narayanan, V. Shmatikov, De-anonymizing social networks, in *2009 30th IEEE Symposium on Security and Privacy* (IEEE, 2009), pp. 173–187.

45. C. Riederer, Y. Kim, A. Chaintreau, N. Korula, S. Lattanzi, Linking users across domains with location data: Theory and validation, in *Proceedings of the 25th International Conference on World Wide Web* (International World Wide Web Conferences Steering Committee, 2016), pp. 707–719.

46. L. Rocher, J. M. Hendrickx, Y.-A. de Montjoye, Estimating the success of re-identifications in incomplete datasets using generative models. *Nat. Commun.* **10**, 3069 (2019).

47. A. Farzanehfar, F. Houssiau, Y.-A. de Montjoye, The risk of re-identification remains high even in country-scale location datasets. *Patterns* **2**, 100204 (2021).

48. A. Cavoukian, D. Castro, in *Big Data and Innovation, Setting The Record Straight: De-Identification Does Work* (Information and Privacy Commissioner, 2014).

49. M. Elliot, E. Mackey, K. O'Hara, *The Anonymisation Decision Making Framework: European Practitioners' Guide* (UK Anonymisation Network, 2020), pp. 78–79.

50. C. Mitchell, J. Ordish, E. Johnson, T. Brigden, A. Hall, *The GDPR and Genomic Data—The Impact of the GDPR and DPA 2018 on Genomic Healthcare and Research* (PHG Foundation, 2020), pp. 51–53.

51. The Instacart online grocery shopping dataset 2017; www.instacart.com/datasets/grocery-shopping-2017 [accessed 16 April 2019.

52. J.-B. Grill, F. Strub, F. Altché, C. Tallec, P. Richemond, E. Buchatskaya, C. Doersch, B. Pires, Z. Guo, M. Azar, B. Piot, K. Kavukcuoglu, R. Munos, M. Valko, Bootstrap your own latent: A new approach to self-supervised learning, in *Neural Information Processing Systems* (Curran Associates Inc., 2020).

53. T. Chen, S. Kornblith, M. Norouzi, G. Hinton, A simple framework for contrastive learning of visual representations, in *International Conference on Machine Learning* (PMLR, 2020), pp. 1597–1607.

54. F. Schroff, D. Kalenichenko, J. Philbin, Facenet: A unified embedding for face recognition and clustering, in *Proceedings of the IEEE conference on computer vision and pattern recognition* (IEEE, 2015), pp. 815–823.

55. L. Alessandretti, P. Sapiezynski, V. Sekara, S. Lehmann, A. Baronchelli, Evidence for a conserved quantity in human mobility. *Nat. Hum. Behav.* **2**, 485–491 (2018).

56. M. C. Gonzalez, C. A. Hidalgo, A.-L. Barabasi, Understanding individual human mobility patterns. *Nature* **453**, 779–782 (2008).

57. C. Dwork, Differential privacy: A survey of results, in *International Conference on Theory and Applications of Models of Computation* (Springer, 2008), pp. 1–19.

58. Location Guard; https://github.com/chatziko/location-guard/ [accessed 21 July 2020].

59. Geoprivacy Plugin: A set of location privacy tools for geographic data; https://diuke.github.io/GeoPrivPlugin/ [accessed 28 March 2021].

60. M. E. Andrés, N. E. Bordenabe, K. Chatzikokolakis, C. Palamidessi, Geo-indistinguishability: Differential privacy for location-based systems, in *Proceedings of the 2013 ACM SIGSAC Conference on Computer & Communications Security* (Association for Computing Machinery, 2013) pp. 901–914.

61. K. Chatzikokolakis, C. Palamidessi, M. Stronati, A predictive differentially-private mechanism for mobility traces, in *International Symposium on Privacy Enhancing Technologies Symposium* (Springer, 2014), pp. 21–41.

62. K. Chatzikokolakis, E. Elsalamouny, C. Palamidessi, Efficient utility improvement for location privacy. *Proc. Priv. Enh. Technol.* **2017**, 308–328 (2017).

63. S. Oya, C. Troncoso, F. Pérez-González, Is geo-indistinguishability what you are looking for? in *Proceedings of the 2017 on Workshop on Privacy in the Electronic Society* (Association for Computing Machinery, 2017), pp. 137–140.

64. M. Alvim, K. Chatzikokolakis, C. Palamidessi, A. Pazii, Metric-based local differential privacy for statistical applications, in *31st Computer Security Foundations Symposium (CSF 2018)* (IEEE Computer Society, 2018), pp. 262–267.

65. M. Cunha, R. Mendes, J. P. Vilela, Clustering Geo-indistinguishability for privacy of continuous location traces, in *2019 4th International Conference on Computing, Communications and Security (ICCCS)* (IEEE, 2019), pp. 1–8.

66. Data Protection Working Party Article 29, Opinion 05/2014 on Anonymisation Techniques; https://ec.europa.eu/justice/article-29/documentation/opinion-recommendation/files/2014/wp216_en.pdf [accessed 28 March 2021].

67. T. T. Tanimoto, *Elementary Mathematical Theory of Classification and Prediction* (International Business Machines Corporation, 1958).

68. A. Cecaj, M. Mamei, F. Zambonelli, Re-identification and information fusion between anonymized CDR and social network data. *J. Ambient Intell. Humaniz. Comput.* **7**, 83–96 (2016).

69. L. Rossi, M. Musolesi, It's the way you check-in: Identifying users in location-based social networks, in *Proceedings of the Second ACM Conference on Online Social Networks* (Association for Computing Machinery, 2014), pp. 215–226.

70. C. Y. T. Ma, D. K. Y. Yau, N. K. Yip, N. S. V. Rao, Privacy vulnerability of published anonymous mobility traces, in *Proceedings of the 16th Annual International Conference on Mobile Computing and Networking* (IEEE, 2010), pp. 185–196.

71. S. Gambs, M.-O. Killijian, M. Núñez del Prado Cortez, De-anonymization attack on geolocated data. *J. Comput. Syst. Sci.* **80**, 1597–1614 (2014).

72. Y. De Mulder, G. Danezis, L. Batina, B. Preneel, Identification via location-profiling in GSM networks, in *Proceedings of the 7th ACM Workshop on Privacy in the Electronic Society* (Association for Computing Machinery, 2008), pp. 23–32.

73. F. M. Naini, J. Unnikrishnan, P. Thiran, M. Vetterli, Where you are is who you are: User identification by matching statistics. *IEEE Trans. Inf. Forensics Secur.* **11**, 358–372 (2015).

74. A. Bhattacharyya, On a measure of divergence between two statistical populations defined by their probability distributions. *Bull. Calcutta Math. Soc.* **35**, 99–109 (1943).

75. D. L. Donoho, For most large underdetermined systems of linear equations the minimal ℓ_1-norm solution is also the sparsest solution. *Commun. Pure Appl. Math.* **59**, 797–829 (2006).

76. A. Singhal, Modern information retrieval: A brief overview. *IEEE Data Eng. Bull.* **24**, 35–43 (2001).

77. A. van den Oord, Y. Li, O. Vinyals, Representation learning with contrastive predictive coding. arXiv:1807.03748 [cs.LG] (10 July 2018).

78. V. Mnih, N. Heess, A. Graves, K. Kavukcuoglu, Recurrent models of visual attention, in *Proceedings of the 27th International Conference on Neural Information Processing Systems-Volume 2* (MIT Press, 2014), pp. 2204–2212.

79. D. Bahdanau, K. H. Cho, Y. Bengio, Neural machine translation by jointly learning to align and translate, in *3rd International Conference on Learning Representations* (ICLR, 2015).

80. J. Chorowski, R. J. Weiss, S. Bengio, A. van den Oord, Unsupervised speech representation learning using wavenet autoencoders. *IEEE/ACM Trans. Audio, Speech, Language Process.* **27**, 2041–2053 (2019).

81. M. I. M. Boorstein, A. Shin, "Top US catholic church official resigns after cell phone data used to track him on grindr and to gay bars," *Washington Post*, 21 July 2021; www.washingtonpost.com/religion/2021/07/20/bishop-misconduct-resign-burrill/ [accessed 28 July 2021].

82. Data protection act 2018, section 171 on the re-identification of de-identified personal data; www.legislation.gov.uk/ukpga/2018/12/section/171 [accessed 28 July 2021].

83. M. Gramaglia, M. Fiore, On the anonymizability of mobile traffic datasets. arXiv:1501.00100 [cs.CY] (31 December 2014).

84. A. Oehmichen, S. Jain, A. Gadotti, Y.-A. de Montjoye, OPAL: High performance platform for large-scale privacy-preserving location data analytics, in *2019 IEEE International Conference on Big Data (Big Data)* (IEEE, 2019), pp. 1332–1342.

85. E. J. Williamson, A. J. Walker, K. Bhaskaran, S. Bacon, C. Bates, C. E. Morton, H. J. Curtis, A. Mehrkar, D. Evans, P. Inglesby, J. Cockburn, H. I. McDonald, B. MacKenna, L. Tomlinson, I. J. Douglas, C. T. Rentsch, R. Mathur, A. Y. S. Wong, R. Grieve, D. Harrison, H. Forbes, A. Schultze, R. Croker, J. Parry, F. Hester, S. Harper, R. Perera, S. J. W. Evans,

L. Smeeth, B. Goldacre Factors associated with COVID-19-related death using OpenSAFELY. *Nature* **584**, 430–436 (2020).

86. Z. Anesbury, M. Nenycz-Thiel, J. Dawes, R. Kennedy, How do shoppers behave online? An observational study of online grocery shopping. *J. Consum. Behav.* **15**, 261–270 (2016).

87. D. Wang, D. Pedreschi, C. Song, F. Giannotti, A.-L. Barabasi, Human mobility, social ties, and link prediction, in *Proceedings of the 17th ACM SIGKDD International Conference on Knowledge Discovery and Data Mining* (Association for Computing Machinery, 2011), pp. 1100–1108.

88. C. Song, Z. Qu, N. Blumm, A.-L. Barabási, Limits of predictability in human mobility. *Science* **327**, 1018–1021 (2010).

89. H. Robinson, F. Dall'Olmo Riley, R. Rettie, G. Rolls-Willson, The role of situational variables in online grocery shopping in the UK. *Mark. Rev.* **7**, 89–106 (2007).

90. C. Hand, F. Dall'Olmo Riley, P. Harris, J. Singh, R. Rettie, Online grocery shopping: The influence of situational factors. *Eur. J. Mark.* **43**, 1205–1219 (2009).

91. G. Biau, L. Devroye, *Lectures on the Nearest Neighbor Method* (Springer, 2015), vol. 246.

92. K. Q. Weinberger, L. K. Saul, Distance metric learning for large margin nearest neighbor classification. *J. Mach. Learn. Res.* **10**, 207–244 (2009).

93. J. Goldberger, G. E. Hinton, S. Roweis, R. R. Salakhutdinov, Neighbourhood components analysis. *Adv. Neural Inf. Process. Syst.* **17**, 513–520 (2004).

94. D. R. Cox, V. Isham, *Point Processes* (CRC Press, 1980), vol. 12.

95. D. P. Kingma, J. Ba, Adam: A method for stochastic optimization, in *ICLR (Poster)* (2015).

96. P. E. McKight, J. Najab, Kruskal-Wallis test, in *The Corsini Encyclopedia of Psychology* (John Wiley & Sons, 2010), pp. 1–1.

97. L. van der Maaten, G. Hinton, Visualizing data using t-SNE. *J. Mach. Learn. Res.* **9**, 2579–2605 2008.

98. A.-L. Barabasi, The origin of bursts and heavy tails in human dynamics. *Nature* **435**, 207–211 (2005).

99. H. W. Kuhn, The Hungarian method for the assignment problem. *Nav. Res. Logist. Q.* **2**, 83–97 (1955).

100. T. Murakami, Expectation-maximization tensor factorization for practical location privacy attacks. *Proc. Priv. Enh. Technol.* **2017**, 138–155 (2017).

101. F. J. Massey Jr., The Kolmogorov-Smirnov test for goodness of fit. *J. Am. Stat. Assoc.* **46**, 68–78 (1951).

102. B. Braden, The surveyor's area formula. *Coll. Math. J.* **17**, 326–337 (1986).

103. G. Voronoi, Nouvelles applications des paramètres continus à la théorie des formes quadratiques. premier mémoire. sur quelques propriétés des formes quadratiques positives parfaites. *J. Reine Angew. Math.* **1908**, 97–102 (1908).
